# Topology Effects in Molecular Organic Electronic Materials: Pyrene and Azupyrene**

**DOI:** 10.1002/cphc.202100222

**Published:** 2021-05-07

**Authors:** Benedikt P. Klein, Lukas Ruppenthal, Samuel J. Hall, Lars E. Sattler, Sebastian M. Weber, Jan Herritsch, Andrea Jaegermann, Reinhard J. Maurer, Gerhard Hilt, J. Michael Gottfried

**Affiliations:** ^1^ Fachbereich Chemie Philipps-Universität Marburg Hans-Meerwein-Straße. 4 35032 Marburg Germany; ^2^ MAS Centre for Doctoral Training Senate House University of Warwick Gibbet Hill Road Coventry CV4 7AL United Kingdom; ^3^ Department of Chemistry and Centre for Scientific Computing University of Warwick Gibbet Hill Road Coventry CV4 7AL United Kingdom; ^4^ Institut für Chemie Carl von Ossietzky Universität Oldenburg Carl-von-Ossietzky-Straße 9–11 26111 Oldenburg Germany

**Keywords:** organic electronics, topologic design, aromaticity, photoelectron spectroscopy, density functional calculations

## Abstract

Pyrene derivatives play a prominent role in organic electronic devices, including field effect transistors, light emitting diodes, and solar cells. The flexibility in the desired properties has previously been achieved by variation of substituents at the periphery of the pyrene backbone. In contrast, the influence of the topology of the central π‐electron system on the relevant properties such as the band gap or the fluorescence behavior has not yet been addressed. In this work, pyrene is compared with its structural isomer azupyrene, which has a π‐electron system with non‐alternant topology. Using photoelectron spectroscopy, near edge X‐ray absorption fine structure spectroscopy, and other methods, it is shown that the electronic band gap of azupyrene is by 0.72 eV smaller than that of pyrene. The difference of the optical band gaps is even larger with 1.09 eV, as determined by ultraviolet–visible absorption spectroscopy. The non‐alternant nature of azupyrene is also associated with a more localized charge distribution. Further insight is provided by density functional theory (DFT) calculations of the molecular properties and *ab initio* coupled cluster calculations of the optical transitions. The concept of aromaticity is used to interpret the major topology‐related differences.

## Graphical abstract

**Topological Tuning**: Molecular organic materials for application in organic electronics can be tailored by topological design of their π‐electron systems, instead of conventional tailoring by peripheral substituents. This is shown by a multi‐technique comparison of pyrene with its topological isomer azupyrene. The non‐alternant topology of azupyrene causes a more localized charge distribution, smaller electronic and optical gaps, and different fluorescence behavior.

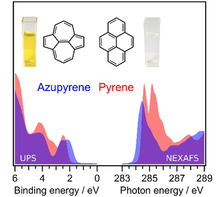

## 1 Introduction

The polycyclic aromatic hydrocarbon pyrene serves as an important building block for semiconductors used in organic electronic devices.^[1]^ Pyrene‐based materials have found application in organic light emitting diodes (OLEDs),^[2]^ organic field effect transistors (OFETs),^[3]^ and organic solar cells (OSCs).^[4]^ One of the most important driving forces enabling the use of pyrene over a wide range of applications is the possible functionalization of its molecular backbone.^[1]^ Accordingly, previous research in the field has focused on modifying the properties of pyrene by introducing substituents at the periphery of the molecule[^2^, ^3^, ^5^] or by incorporating pyrene units into semiconducting polymers.[^4^, ^6^] In contrast, the influence of the topology of the π‐electron system on the properties of pyrene has not yet been addressed.

During the past years, the importance of topology for the molecular properties of organic semiconductors has been increasingly recognized. The inherent promise for performance improvements resulted in a revived interest in non‐alternant π‐electron systems and first efforts to use the related molecules in (opto)electronic devices have been made.^[7]^ However, most of the related studies focus on azulene and its derivatives.^[8]^

**Figure 1:**
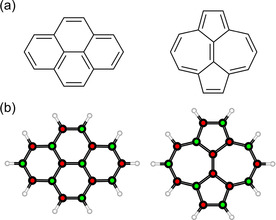
(a) Lewis formulae of pyrene (left) and azupyrene (right). (b) DFT optimized ball‐and‐stick models showing the alternant nature of pyrene (left) and the non‐alternant nature of azupyrene (right). Only in the case of pyrene can the atoms be alternatingly labelled (red/green), while this is not possible for azupyrene.

In this study, we explore the influence of the π‐topology on the properties of polycyclic aromatic hydrocarbons by comparing pyrene (Figure 1, left) with its isomer azupyrene (Figure 1, right). Pyrene has an alternant π‐electron system, which means that the atoms of the π‐system can be classified alternatingly into two exclusive sets (red and green in Figure 1), such that direct connections occur only between atoms of different sets. For azupyrene, such a distribution is not possible, therefore the topology of its π‐system is called non‐alternant.^[9]^ This seemingly abstract difference between alternant and non‐alternant isomers has important practical implications. For example, it can explain why azulene is blue, while its isomer naphthalene is colorless.^[10]^ Additionally, azupyrene is interesting as a molecular model for the Stone‐Wales defect in graphene.^[11]^ In on‐surface synthesis, both pyrene and azupyrene are small prototypical building blocks for alternant and non‐alternant nanographenes.^[12]^

Azupyrene was first synthesized and characterized in 1968^[13]^ and some investigations including ultraviolet‐visible (UV/Vis) spectroscopy,^[14]^ infrared (IR) spectroscopy^[13]^ and nuclear magnetic resonance (NMR) spectroscopy[^13^, ^14c^] measurements were already reported in the literature. Azupyrene was also mentioned in some basic theoretical investigations,^[15]^ and some general work on aromaticity including the harmonic oscillator model of aromaticity (HOMA).^[16]^

Here, we present the first study in which photoelectron spectroscopy and X‐ray absorption spectroscopy are used to directly probe the valence electronic structure in application‐related polycrystalline thin films of pyrene and azupyrene. The thereby obtained electronic band gaps are compared with the optical band gaps, as determined by UV/Vis spectroscopy. We use complementary quantum theoretical calculations on the basis of density functional theory (DFT) and the coupled cluster (CC) approximation to gain a deeper understanding of the electronic structure and observed optical and electronic transitions in UV/Vis as well as in fluorescence spectroscopy. Furthermore, we discuss the results of NMR and IR spectroscopy together with an analysis according to the HOMA approach.

The theoretical methods were always employed on the gas‐phase molecules to have the same reference for experiments performed on thin films and on solutions of the molecules. The gas phase calculations additionally offer a more accessible interpretation using the molecular electronic states. However, we performed additional calculations for the molecular crystals with periodic boundary conditions to gauge the influence of intermolecular interactions, which are present in the experiments involving thin films, on the electronic properties.

Our study reveals and illustrates the striking influence of the π‐electron system‘s topology on the electronic properties. The non‐alternant molecule shows significantly lower electronic and optical band gaps and we found strong indications for lower electron‐hole binding energies, which can facilitate electron‐hole separation. Applying these results in the form of topological design^[17]^ to the development of new materials for organic electronic devices can open up a new pathway for tuning their properties in a systematic manner.

## 2 Results and Discussion

### 2.1 Preparation of the Polycrystalline Thin Films

To gain insight into the electronic structure of azupyrene and pyrene, thin polycrystalline films of the molecules were prepared and studied under ultra‐high vacuum (UHV) conditions. This approach ensured a high sample cleanliness and enabled the application of X‐ray and UV photoelectron spectroscopy (XPS/UPS) as well as near edge X‐ray absorption fine structure (NEXAFS) spectroscopy. The thin films were prepared on a single crystalline Cu(111) substrate by vapor deposition in ultra‐high vacuum. The thickness of the polycrystalline films was chosen large enough (>30 nm) to ensure that the contribution of the molecules with direct contact to the copper substrate to the total signal was negligible. In these films, the molecules have no preferential orientation, as was confirmed by the lack of linear dichroism in angle‐dependent NEXAFS measurements.

### 2.2 X‐ray Photoelectron Spectroscopy

**Figure 2:**
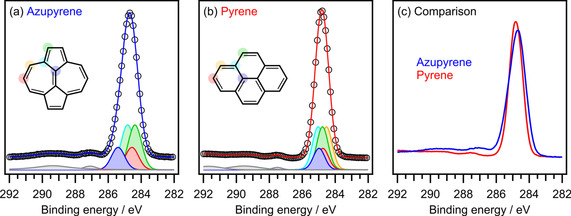
C1s XP spectra (Al‐K_α_, 1486.6 eV) of azupyrene (a) and pyrene (b). The open circles are the experimental spectra, while blue and red lines represent highly restricted fits based on theoretical core‐level binding energies calculated by DFT. The individual DFT‐calculated component peaks for the symmetry equivalent carbon atoms are colored according to the colored circles in structural formulae. The gray peaks are attributed to shake‐up satellites. The DFT peak positions were calculated with a PBE exchange‐correlation functional. For further details, see the SI. (c) Direct comparison of the experimental spectra (here displayed as line graphs to highlight the different peak shapes).

Figure 2 shows X‐ray photoelectron (XP) spectra of the azupyrene and pyrene films. The C1s signals are centered at 284.66 eV for azupyrene and 284.81 eV for pyrene with respect to the Fermi energy *E*_F_. The azupyrene peak shows a larger width (full width at half maximum, FWHM=1.16 eV) and is slightly asymmetric with a shoulder at the high binding energy side. The pyrene peak is symmetric and has a smaller width (FWHM=0.94 eV). The differences in peak shape and peak width can be explained with the help of DFT calculations, which provide the binding energies for the individual carbon atoms in the molecules. For this purpose, the core‐electron binding energies were calculated with a localized core‐hole in a ΔSCF scheme and compared to the experimental data by way of a fitting procedure. In this procedure, the relative binding energies and relative intensities (given by the number of symmetry equivalent carbon atoms) were taken as fixed parameters forming the overall theoretical C 1s peak of the molecule as sum over the equivalent carbon sub peaks, each represented by a pseudo‐Voigt peak.^[18]^ The single‐peak‐width and the Gaussian‐to‐Lorentzian ratio were constricted to be equal for all sub peaks and were parameters of the fit procedure together with the overall intensity and a global energy shift. As can be seen in Figure 2a,b, the agreement between experiment and theory is remarkably good, allowing us to explain the peak shape based on the theoretical model. According to this analysis, the asymmetry of the azupyrene peak is caused by the more distinctly different chemical environments of its carbon atoms. Especially the signal related to the carbon atoms in the central bridge (blue in Figure 2a,b) is shifted by quite a large margin to higher energies, explaining the asymmetry and the high energy tail of the azupyrene peak. Related questions of the different charge distributions in these molecules will be discussed further below.

### 2.3 Analysis of the Valence Electronic Structure

**Figure 3:**
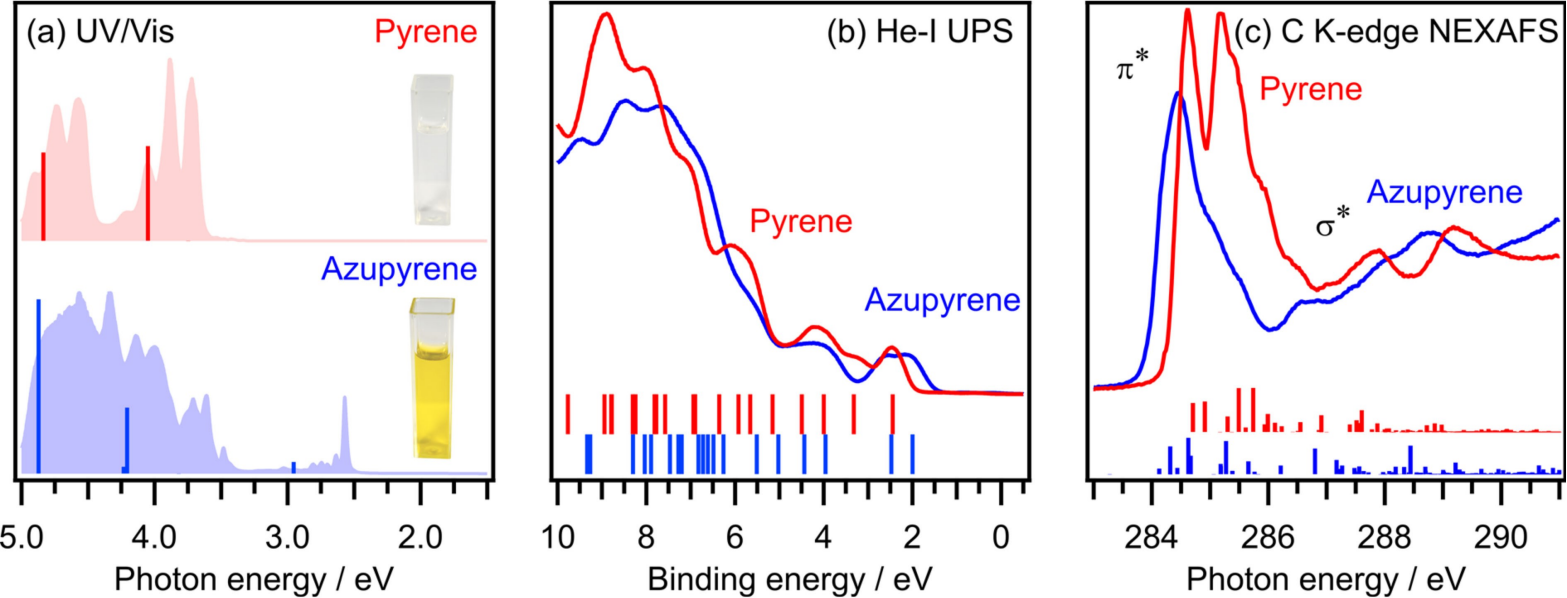
(a) UV/Vis spectra of azupyrene (blue shaded) and pyrene (red shaded) recorded for 0.1 mm solutions in cyclohexane. Pictures of these solutions are displayed next to the corresponding spectra. The vertical lines in the spectra indicate transition energies and probabilities calculated with the CC2 method. (b) He−I UPS spectra of the polycrystalline thin films, vertical lines represent the Kohn‐Sham orbital energies obtained with the B3LYP exchange correlation functional. (c) C K‐edge NEXAFS spectra of the polycrystalline thin films, vertical lines represent transition energies and probabilities calculated with the PBE functional. The calculated data for the UPS and NEXAFS spectra was rigidly shifted to match the experimental energy scale. The calculated NEXAFS transitions have already been published in context of method development.^[19]^

Azupyrene with its non‐alternant π‐electron system has a much smaller optical gap than its alternant isomer pyrene. This is apparent in the intense yellow color displayed by solutions of azupyrene, whereas solutions of pyrene are colorless (See insets in Figure 3a). In agreement with the yellow color of azupyrene, the UV/Vis spectrum (Figure 3a) shows an absorption peak for azupyrene at a photon energy of 2.6 eV, in the blue part of the visible range. Pyrene has its lowest energy peak at a much higher photon energy of 3.7 eV, well outside the visible range. The resulting optical excitation energies, determined from the rising edge of the peak, are 2.54 eV for azupyrene and 3.63 eV for pyrene, yielding a difference of 1.09 eV. Our *ab initio* calculations within the second‐order approximate coupled‐cluster (CC2) approach (see vertical lines in Figure 3a) overestimate the absolute excitation energies for both molecules. However, the calculations yield a difference in transition energies of 1.10 eV between azupyrene and pyrene, in excellent agreement with the experiment. It should be noted that while the lowest energy excitation corresponds to the transition from the highest occupied to the lowest unoccupied molecular orbital (HOMO→LUMO) in pyrene, this transition is symmetry forbidden for azupyrene and the peak in the visible range has to be attributed to the transition into a higher lying unoccupied orbital (HOMO→LUMO+1).

The optical gap determined by UV/Vis spectroscopy must be distinguished from the electronic gap, which is defined as the difference between the electron affinity and the ionization potential. While strictly speaking not equivalent, this is often approximated with the DFT orbital energy difference between HOMO and LUMO. We were able to obtain direct information about the electronic gap of the two molecules by UPS (Figure 3b) and NEXAFS (Figure 3c), which probe the occupied and unoccupied states, respectively.

The peaks observed in the UV photoelectron (UP) spectra (Figure 3b) can be discussed in correspondence to Kohn‐Sham orbital energies obtained by gas‐phase DFT calculations. The comparison of gas‐phase calculations to spectroscopic measurements of molecular crystals is a common approximation and is justified by the fact that the weak intermolecular van der Waals interactions in the solid film usually have only a very small influence on the electronic states.^[20]^ We also performed periodic calculations of the molecular crystals to prove this point for our systems. The results of these calculations are shown in Figure S1 of the supporting information (SI).

**Figure 4:**
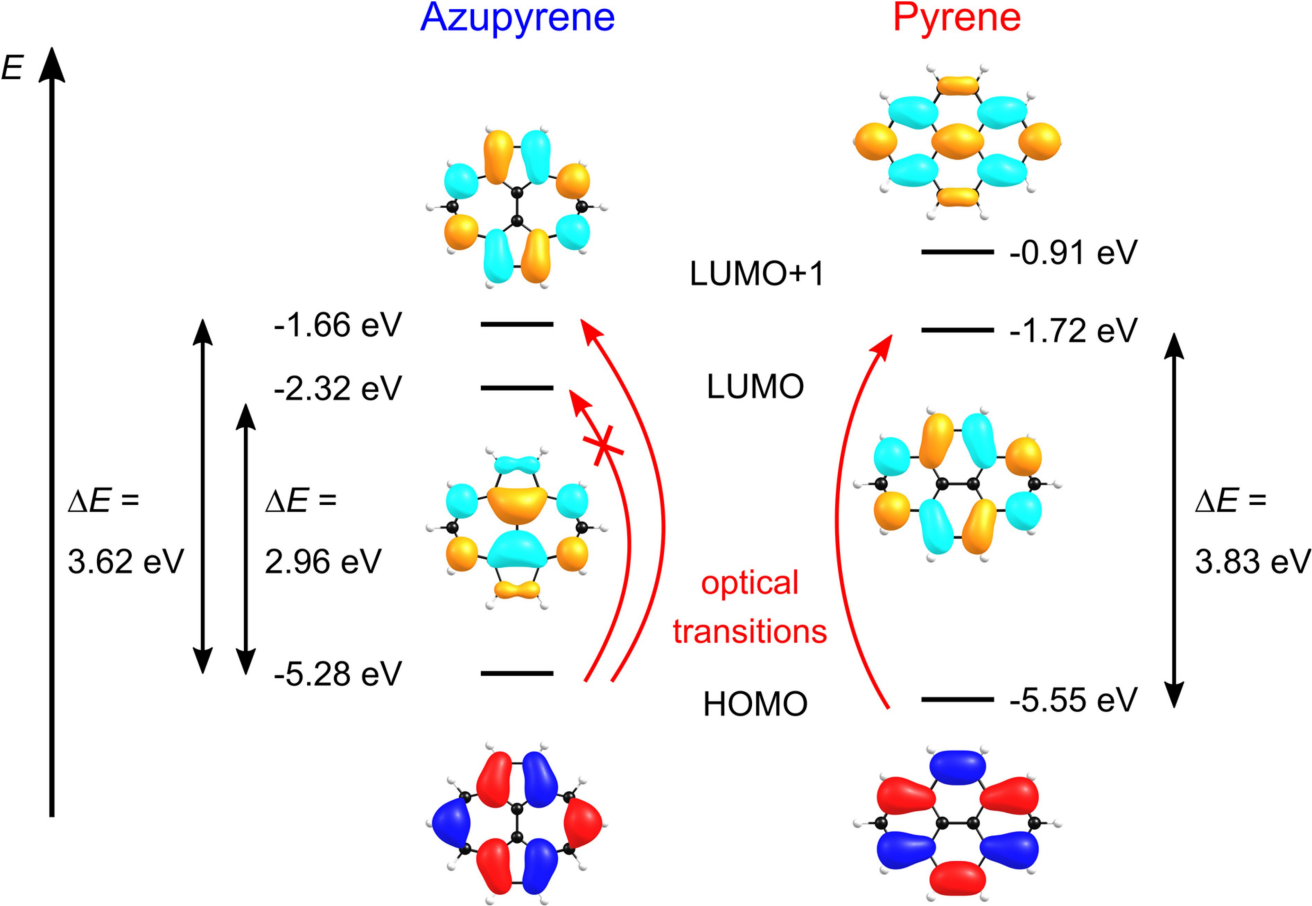
(a) Molecular frontier orbitals and their energies calculated with the B3LYP exchange correlation functional. Left: azupyrene, right: pyrene. The black arrows indicate the electronic band gaps. For azupyrene the electronic band gap is shown including and excluding the LUMO. The red arrows indicate the optical transitions possessing the lowest energy, the HOMO→LUMO transition is dipole‐forbidden for azupyrene. A more detailed Jablonski scheme is shown in the SI, Figure S5. Molecular wave functions are shown for HOMO, LUMO, and LUMO+1 of both molecules (iso‐value: 0.03).

Experimentally, the first electron removal energy, which we can associate with the highest occupied state of azupyrene, is situated at a binding energy of 2.10 eV, which is by 0.35 eV higher in energy for pyrene (2.45 eV). The difference of 0.35 eV is in reasonable agreement with the energy difference of the HOMOs of 0.27 eV provided by DFT calculations. The DFT orbital energy scheme is plotted together with depictions of the orbital wave functions in Figure 4.

The carbon K‐edge NEXAFS spectra (Figure 3c) show intense π* resonances at low photon energy (284 to 287 eV) and less intense σ* resonances at higher photon energies (>287 eV). The first π* resonance of pyrene has two maxima, which are caused by the C1s→LUMO and C1s→LUMO+1 excitations, as was ascertained by MO‐projected NEXAFS simulations (see Figure S2 in the SI and Ref. [19]). For azupyrene, the individual excitations in the first peak are not resolved, because the corresponding LUMO, LUMO+1 and LUMO+2 orbitals are too close in energy. For both molecules, the MO‐projected NEXAFS simulations show that the leading edge of the adsorption peak is determined by the C1s→LUMO excitation.

The X‐ray absorption edge of azupyrene appears at a lower photon energy by 0.37 eV than that of pyrene, as can be seen in Figure 3c. Since the core‐level energies differ only marginally, it can therefore be concluded that the LUMO of azupyrene is lower in energy. Theory also predicts the LUMO of azupyrene to be lower in energy, here the magnitude is 0.60 eV.

Combination of the experimental differences of electron addition and removal energies from UPS and NEXAFS data indicate that the electronic band gap is 0.72 eV (=0.37 eV+0.35 eV) smaller for azupyrene than for pyrene. For comparison, the calculations yield a value of 0.87 eV for the difference in electronic band gap, which is in good agreement with the experimental finding. In addition, the calculations can provide the absolute electronic band gaps, which are not accessible by the experimental techniques at our disposal. The calculated absolute electronic band gaps are 2.96 eV and 3.83 eV for azupyrene and pyrene, respectively. These values can be compared to the optical band gaps of 2.54 eV for azupyrene and 3.63 eV for pyrene, as determined by UV/Vis, yielding a deviation between (experimental) optical and (calculated) electronic band gaps of 0.42 eV for azupyrene and 0.20 eV for pyrene.

The deviation between the electronic and the optical gap for azupyrene is in fact even larger due to the symmetry selection rules at play. The HOMO→LUMO dipole transition is symmetry forbidden for azupyrene and therefore the LUMO energy does not control the optical band gap. (It should be noted that the HOMO→LUMO transition might still be responsible for a very weak signal at about 1.8 eV as shown in Figure S3 in the SI, which is neglected for the following calculations.) For pyrene, the HOMO→LUMO transition is allowed and determines the optical band gap. If the difference between the optical band gap and the electronic band gap is to be calculated in a meaningful manner using electronic “frozen orbital” transition energies, the LUMO has to be excluded for azupyrene. Accordingly, the DFT‐calculated electronic band gap of azupyrene increases from 2.96 eV to 3.62 eV. With this value the difference between the DFT‐based “frozen orbital” electronic band gap and the (experimental) optical band gap is 1.08 eV (=3.62 eV−2.54 eV) for azupyrene, while the same difference is only 0.20 eV (=3.83 eV−3.63 eV) for pyrene.

The large deviation between the electronic and optical band gap for azupyrene is a direct consequence of its non‐alternant π‐electron system. This topological property of azupyrene leads to a localization of the molecular orbitals. When an electron is excited into a higher lying orbital, then its initial and final orbitals can be located in different parts of the molecule, thus decreasing electron‐electron repulsion in the excited state and lowering the transition energy. This explanation was already used to explain the color of the prototypical non‐alternant molecule azulene.^[10]^ The spatial separation of the excited electron from its orbital of origin (the hole left behind upon excitation) suggests a small electron‐hole binding energy in azupyrene. Therefore, it is reasonable to assume that electron and hole, i. e., positive and negative charge carriers, can more easily be separated in azupyrene‐based than in pyrene‐based organic electronics devices.

For illustration, the frontier orbitals of azupyrene are shown in Figure 4: the HOMO is partly localized at the tip‐atoms of the 7‐membered rings and has vanishing coefficients at the neighboring atoms and the central C_2_‐unit, whereas the LUMO+1 shows a reversed localization with a nodal plane running through the tip atoms of the 7‐membered ring (see Figure 4, left). In contrast, the HOMO and LUMO of pyrene are linked by the Coulson‐Rushbrooke pairing theorem^[21]^ and thus are localized on the same carbon atoms (see Figure 4, right).

Thus, the lower band gap of azupyrene is caused by its non‐alternant nature in two ways: (i) the “frozen orbital” electronic band gap is smaller, because the HOMO is higher in energy and the LUMO is lower in energy and (ii) the reduced electron‐electron repulsion in the excited state leads to an even smaller optical band gap.

The fact that the HOMO→LUMO transition is forbidden for azupyrene has further consequences for its fluorescence spectrum. Indeed, no substantial fluorescence could be detected for azupyrene (see Figure S4 of the SI). Adhering to Kasha's rule, the azupyrene molecule would relax non‐radiatively to the lowest excited singlet state S_1_, where one electron resides in the former LUMO. As the LUMO→HOMO fluorescence transition is dipole forbidden, the molecule has then to relax by non‐radiative channels to the ground state. One special factor that could contribute to the non‐radiative decay possibilities is the so‐called Stone‐Wales rearrangement, which may be mediated by the excited state.^[22]^ We found no indications of fluorescence from higher states or phosphorescence decay. However, these processes might still occur with low probabilities. Further discussion and a Jablonski‐diagram can be found in the SI (Figure S5). The lack of fluorescence may be a major advantage for the application of azupyrene in organic solar cells.

### 2.4 Analysis of the Molecular Charge Distribution and IR Spectroscopy

**Figure 5:**
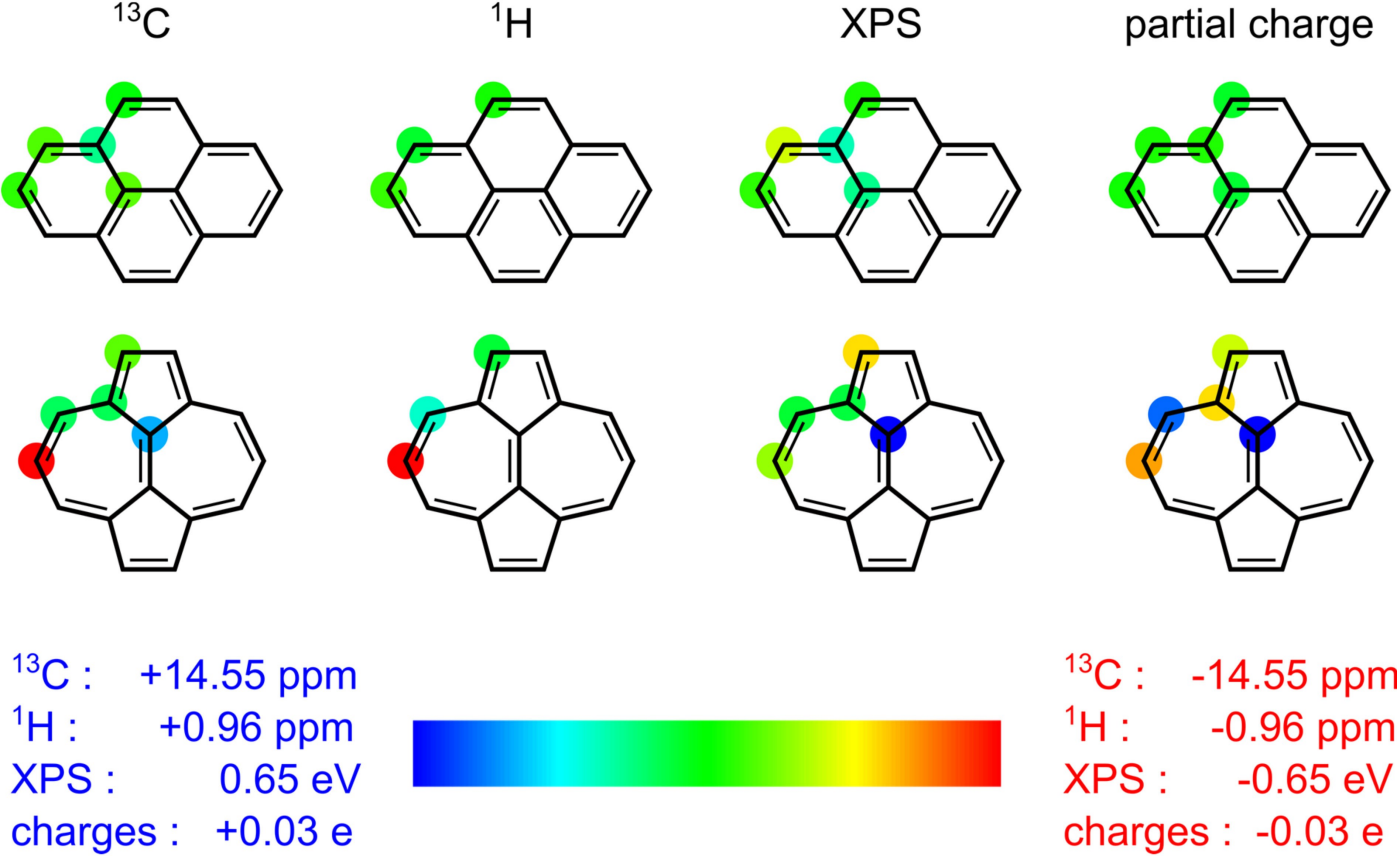
Comparison of experimental and theoretical results correlated to the charge distribution in the molecules. All methods are sensitive to the electron density at the carbon atoms. Red means high electron density (negative charge), blue means low electron density (positive charge) at the respective atom. NMR and XPS shifts are given relative to the averaged shift for the respective molecule.

The non‐alternant nature of azupyrene also influences the charge distribution in the molecule, as was already indicated by the XPS shifts discussed above (Figure 2). Figure 5 correlates the results of different experimental and theoretical methods sensitive to the local electron density at each atom. NMR shifts are well known to be strongly correlated to the electron density at the respective atom.^[23]^ The ^13^C and ^1^H NMR spectra recorded of pyrene and azupyrene in solution are shown in Figure S6 of the SI together with DFT calculations of the NMR‐shifts and the resulting peak assignment. In addition to the experimental ^13^C and ^1^H NMR shifts, Figure 5 contains the XPS shifts, which were calculated by DFT but substantiated by the agreement with the experimental data (Figure 2a,b), and the partial charges as calculated by means of the Hirshfeld charge analysis.^[24]^

For pyrene, all methods indicate an overall uniform charge distribution, as is expected for a π‐electron system with alternant topology. In the case of azupyrene, the charge distribution is less uniform and shows the following trends: (i) positive charge accumulation at the central two carbon atoms and (ii) negative charge accumulation at the tip atoms of the 7‐membered rings. These trends are evident for all methods to varying degrees. These results indicate that the small change in topology, which distinguishes pyrene from azupyrene, has a remarkable influence on the electronic structure as manifested in the electronic and optical band gaps as well as the local charge distribution.

**Figure 6:**
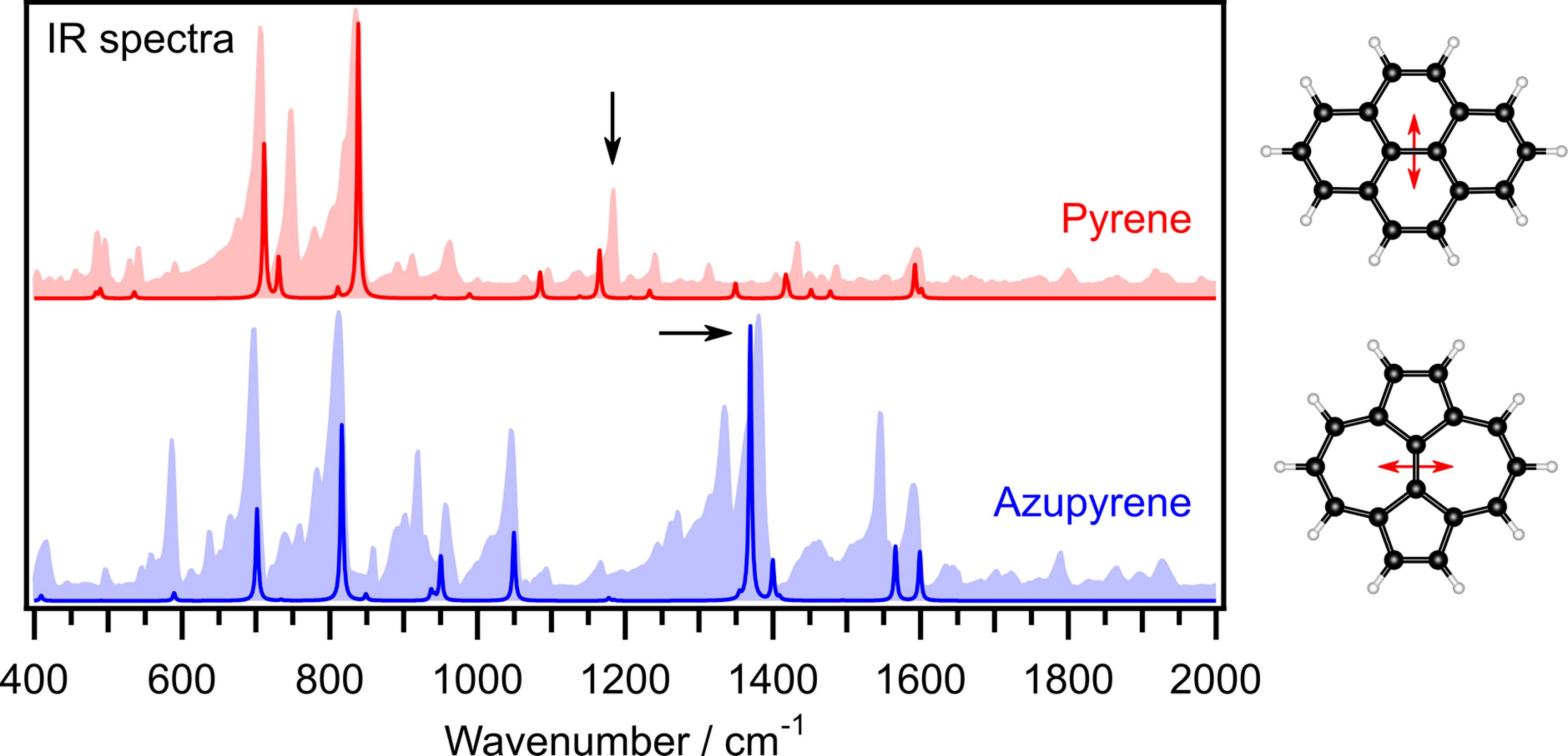
IR spectra of pyrene (red) and azupyrene (blue) compared to the results of DFT calculations (PBE functional). Shaded areas, experimental spectra; lines, calculated vibrational modes and IR‐intensities. Each calculated transition is represented by a Lorentzian line with a FWHM of 4 cm^−1^. The lines belonging to the vibrational modes shown in the right part of the figure are marked by arrows in the graph. Animations of both vibrations are provided with the SI. The spectra were acquired with an attenuated total reflection IR spectrometer using the powdered materials (see the Experimental Section for details).

The topological difference is also manifest in the molecular vibrations, which are experimentally accessible with IR spectroscopy (Figure 6). Azupyrene shows an intense line at 1381 cm^−1^, visible in both the experimental and the simulated spectrum. The corresponding vibrational mode is the vibration of the central C_2_ unit relative to the perimeter of the azupyrene molecule, as visualized in the right part of Figure 6. For pyrene, a similar vibration occurs in the spectrum, but at lower energy (1184 cm^−1^) and with lower intensity. Animated visualizations of both vibrations are provided as supplementary information. The differences between the IR spectra are related to the different intramolecular bond strengths, which in turn reflect the different conjugation mechanisms in the π‐electron systems.

### 2.5 HOMA Analysis

In the following, we will rationalize the effects of the topological difference between pyrene and azupyrene on the π‐electron system using the harmonic oscillator model of aromaticity (HOMA).^[25]^ The HOMA value *H* is defined as
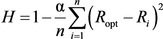



and is related to the deformation energy of a π‐electron system, approximated by a harmonic potential around the ideal aromatic bond length *R*_opt_. In Equation (1), *n* is the number of bonds (with lengths *R_i_*) in the conjugation path. The parameter α is an empirical constant chosen to give *H*=1 for the ideal aromatic system of benzene (with six equal bond lengths) and *H*=0 for the hypothetical Kekulé cyclohexatriene structure of benzene (with three single and three double bonds). The physical meaning of *H* is based on the fact that bonds in a conjugated π‐electron system have lengths between pure σ‐ and π‐bonds, which means that the σ‐bonds are compressed, and the π‐bonds are extended in the final equilibrium structure. Both the σ‐bond compression and the π‐bond extension cost energy. *R*_opt_ represents the optimum bond lengths, at which compression of the typical σ‐bond to the value of *R*_opt_ is equal to the energy of extension of a typical π‐bond to *R*_opt_.^[26]^ For the HOMA values discussed below, we used the bond lengths obtained from the DFT‐optimized structures of the two molecules (PBE functional). The model parameters (*R*_opt_=1.397 Å and α=352.1 Å^−2^) used in Equation 1 were determined from the structures of the free benzene and trans‐butadiene molecules optimized with the same DFT method.

For each molecule with more than one aromatic ring, different possible conjugation paths exist, each of which is characterized by its HOMA value. A high HOMA value indicates that this particular path strongly contributes to the aromatic stabilization. It is also possible to consider all π‐bonds in the molecule; the corresponding HOMA value is called the overall HOMA value *O*. If the conjugation path along the perimeter π‐bonds of the molecule is considered, this is called the perimeter HOMA value *P*. Likewise, the HOMA value *R* for each ring can be calculated separately.

**Figure 7:**
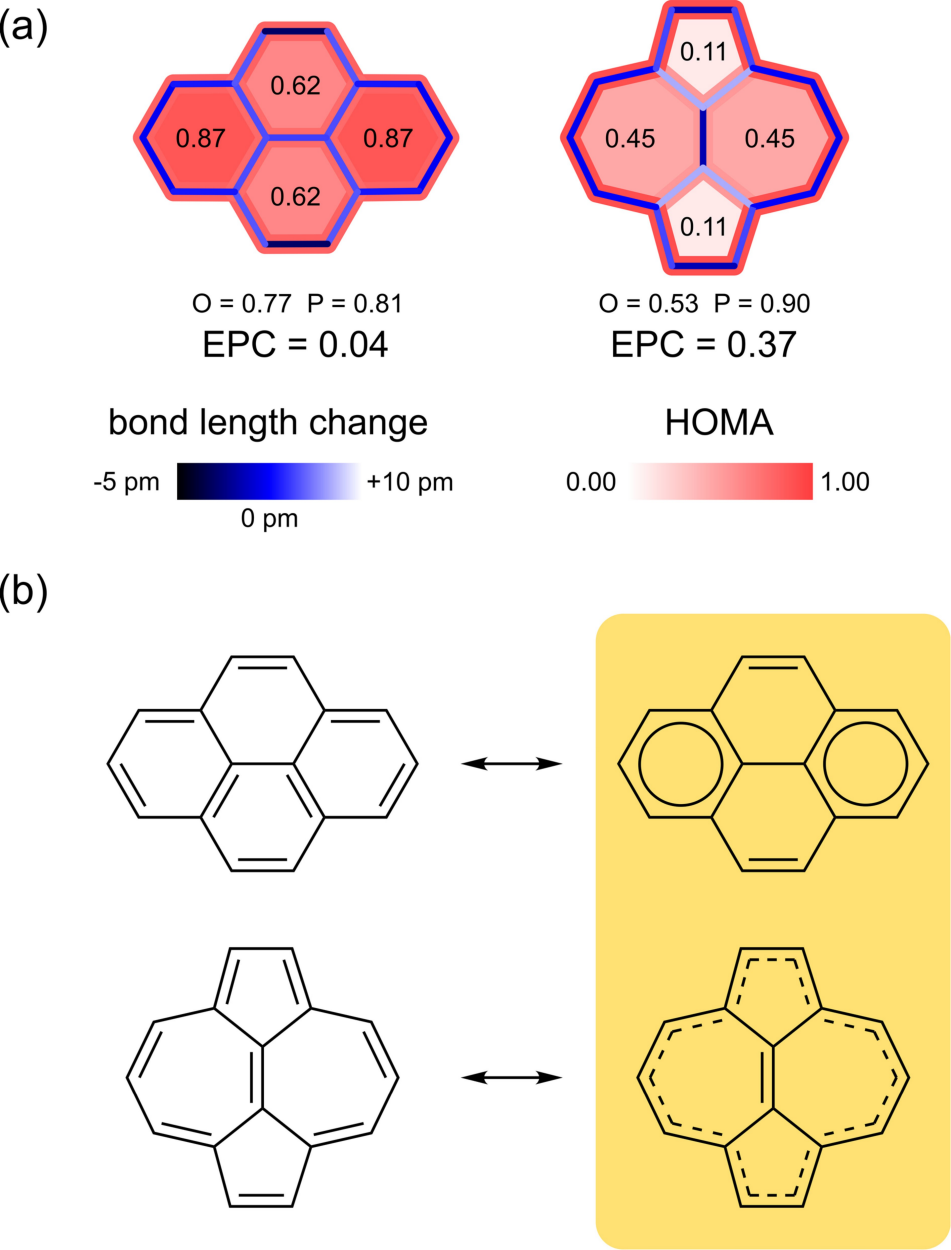
(a) HOMA analysis for azupyrene and pyrene, based on their DFT‐optimized structures (PBE functional). The red‐shaded color scheme indicates the HOMA values according to the provided scale. The fillings represent the HOMA values of the individual rings (*R*). The perimeter bonds are red‐colored according to the perimeter HOMA value (*P*), and the bridging bonds are red‐colored according to the overall HOMA value (*O*). All bonds are additionally colored with a blue color scheme representing the deviation of the bond length from the ideal aromatic bond length *R*_opt_. For the definition of *EPC*, see the text. (b) Comparison of the regular resonance structures with the conjugation patterns determined by the HOMA analysis. The conjugation in pyrene can be described as doubly ethenediyl bridged biphenyl with two Clar sextets, whereas the conjugation in azupyrene is best described as a [14]annulene with an isolated, π‐bonded C_2_ unit in the center.

For convenience, we also calculated the Excess Perimeter Conjugation (*EPC*) value, which is defined as *EPC*=*P−O*.^[27]^ This parameter indicates whether the molecule shows annulenoid character, i. e., the aromatic conjugation is predominantly on the perimeter of the molecule (high *EPC*), or benzenoid character, i. e., the conjugation is distributed over the whole molecule equally (low *EPC*). The results of the HOMA analysis are compiled in Figure 7.

Both molecules comprise 16 π‐electrons. A conjugation pathway including all 16 π‐electrons would be anti‐aromatic, according to Hückel's 4*n* rule.^[28]^ However, for neither molecule a conjugation path with 16 π‐electrons exists and pyrene as well as azupyrene are aromatic, as is proven by the ^1^H‐NMR shifts (see Figure S6). While both molecules are aromatic, the difference between them lies in the different conjugation mechanism leading to the aromatic stabilization.

Pyrene has a small *EPC* value of 0.04, which indicates that no annulenoid conjugation is present. The two apical rings possess higher HOMA values than the two other rings and these rings with the smaller HOMA value have one especially short bond. Both facts indicate that the most fitting description of the pyrene molecule is a biphenyl doubly bridged by ethenediyl units (Figure 7b, right). The two apical rings form Clar sextets (2×6 π‐electrons) and the ethylene bridges form quasi‐isolated double bonds (2×2 π‐electrons). Thus, the 16 π‐electron system of pyrene can be described as a 6π+6π+2π+2π system.

Azupyrene has a large *EPC* value of 0.37, a short central bond (138 pm) with pronounced double‐bond character, and elongated bonds (147 pm) with single‐bond character connecting the central two carbon atoms to the perimeter. These facts agree with the description of azupyrene as a [14]annulene with a central C_2_ bridge that is partly decoupled from the annulene‘s π‐system (Figure 7b, right). In this way, the 16 π‐electrons are divided into 14 π‐electrons on the perimeter and 2 π‐electrons in the quasi‐isolated central double bond, forming two Hückel‐compliant systems (14π+2π system). This is not a trivial finding, because the molecule could also form a 6π+6π+2π+2π system with two negative 6π cyclopentadienyl (Cp^−^) rings bridged by two positive propenediylium‐bridges (Pr^+^). Such a conjugation type is contradicted by the HOMA values and the bond length pattern. In addition, if azupyrene would follow the latter conjugation mechanism, it should possess a strong quadrupole moment (in analogy to the dipole moment of azulene), which is not present in our DFT calculations.

To obtain additional insight into the aromaticity, we also performed calculations using the nucleus‐independent chemical shift (NICS) method,^[29]^ which are presented in detail in Table S7 of the SI. For pyrene, the NICS calculations show a higher grade of aromaticity for the apical rings compared to the lateral rings, in agreement with the HOMA analysis and the model of a doubly ethenediyl bridged biphenyl with two Clar sextets. For azupyrene, the NICS values also show that all rings are aromatic, but the dependence of the NICS values on the ring size makes a further comparison impractical.

## 3 Conclusions

Our systematic multi‐technique comparison of the organic semiconductor pyrene and its non‐alternant isomer azupyrene revealed major topology‐related differences of their electronic properties. UPS and NEXAFS show that the electronic band gap of azupyrene is by 0.72 eV smaller than that of the alternant molecule pyrene, and theory predicts a very similar difference of 0.87 eV. The optical gap for the lowest energy transition is 2.54 eV for azupyrene and 3.63 eV for pyrene, yielding an even larger difference of 1.09 eV. One reason why this difference is larger for azupyrene is due to symmetry selection rules, which result in a forbidden HOMO→LUMO transition and the lack of fluorescence for azupyrene. These special properties of azupyrene's electronic structure are related to the non‐alternant topology of its π‐electron system. The topology of azupyrene also leads to a more localized charge distribution, visible in the experimental XPS and NMR shifts as well as in the DFT‐calculated partial charges, all showing a negative charge surplus at the apices of the 7‐membered rings and positive charge at the central C_2_‐unit. The conjugated system of pyrene is best described as a biphenyl with two ethenediyl bridges, whereas azupyrene is a [14]annulene with a central double‐bonded C_2_ unit. This interpretation follows from HOMA considerations, bond length changes, and the vibrational modes seen in experimental and calculated IR spectra. Overall, we found that the topology of the π‐electron system drastically influences the electronic structure of the two polycyclic aromatic hydrocarbons. Those differences in electronic structure are reflected in the absorption and fluorescence behavior, a lower electron‐hole binding energy, and a lower barrier to electron‐hole separation for azupyrene. The possible application of non‐alternant molecules in organic electronic devices is favored by those differences. We propose the utilization of these results via topological design to tune the properties of organic semiconductors in the search for future materials for organic electronics.

## Experimental Section

### Experimental Methods

The synthesis of azupyrene (dicyclopenta[ef,kl]heptalene) is described in the SI. Pyrene war purchased from Sigma‐Aldrich (purity >99 %). The XPS, UPS and NEXAFS measurements were performed in ultra‐high vacuum systems with base pressures below 2⋅10^−10^ mbar. Azupyrene and pyrene were deposited onto a Cu(111) single crystal substrate with a home‐build line‐of‐sight evaporator after initial freeze‐pump‐thaw cycles of the reservoirs. The thickness of the films was between 30 and 70 nm. The lack of order in the films was proven by the lack of dichroism in NEXAFS measurements. All measurements for the thin films were performed at a temperature of below 200 K to prevent desorption of molecules from the thin film. XPS and UPS were performed with a SPECS PHOIBOS 150 electron energy analyzer equipped with a MCD‐9 multi channeltron detector. For XPS, monochromatic Al‐K_α_ radiation from a SPECS XR 50 M X‐ray anode with a FOCUS 500 monochromator was employed. For UPS, He−I radiation from a SPECS UVS 10/35 gas discharge source was used. NEXAFS measurements were performed at the synchrotron radiation facility BESSY II (Helmholtz‐Zentrum Berlin) using the HE‐SGM dipole beamline. The partial electron‐yield (PEY) detection method was used with a retarding field of −150 V and a channeltron detector voltage of 2.3 keV. UV/Vis spectroscopy was performed at room temperature using a Shimadzu UV‐1650PC double beam spectrophotometer with a halogen and a deuterium lamp as light sources and a silicon photodiode as detector. The molecules were prepared for the UV/Vis measurements as solutions in cyclohexene with a concentration of 0.1 mmol l^−1^; pure cyclohexene served as a reference placed in the reference beam. The IR spectra were recorded at room temperature for the powdered material using a Bruker Tensor IF37 attenuated total reflection IR spectrometer. NMR spectroscopy was performed at room temperature on a Bruker Avance II 300 MHz spectrometer using CD_2_Cl_2_ as a solvent.

### Computational Details

Density‐functional‐theory calculations were performed using the program package Gaussian16^[30]^ and the def2‐TZVPP basis set.^[31]^ For the structural optimization and frequency calculations the generalized gradient approximation (GGA) proposed by Perdew, Burke, and Ernzerhof (PBE)^[32]^ was used as the exchange‐correlation functional in combination with the D3 van‐der‐Waals correction scheme including Becke‐Johnson damping.^[33]^ The electronic properties of the molecules, i. e., Kohn‐Sham orbital eigenenergies and Hirshfeld partial charges,^[24]^ were obtained using the B3LYP hybrid functional.^[34]^ The optical transition energies and intensities were obtained in Turbomole 7.4.^[35]^ The *ab initio* second‐order approximate coupled‐cluster (CC2) calculations^[36]^ were performed on the DFT‐optimized structures using the B3LYP functional and the def2‐TZVPP basis set. Calculations for XPS and NEXAFS simulations were performed using the pseudopotential plane‐wave code CASTEP‐18.1^[37]^ with the PBE functional^[32]^ and a plane‐wave cutoff of 500 eV. The delta self‐consistent field (ΔSCF) method of constraining electronic occupations to resemble full core‐hole excitations was employed to generate XPS shifts. Core‐level spectra were processed using the MolPDOS post‐processing tool in CASTEP.^[38]^ NEXAFS calculations were performed using on‐the‐fly generated ultra‐soft pseudopotentials (USPPs) with the CASTEP module ELNES^[39]^ with the transition‐potential approach,^[40]^ which constrains the occupation of the C 1s initial state orbital to 0.5 and the corresponding Kohn‐Sham eigenenergies are taken to reflect the transition energies in the NEXAFS spectrum. The intensities for each transition are calculated as the magnitude of the transition dipole matrix elements. The overall spectrum is obtained by shifting the atomic NEXAFS contributions according to their ΔSCF binding energies following the so‐called ΔIP‐TP method.[^19^, ^40a^]

## Supporting Information

Supporting Information is available from the Wiley Online Library or from the author.

## Conflict of interest

The authors declare no conflict of interest.

## Supporting information

As a service to our authors and readers, this journal provides supporting information supplied by the authors. Such materials are peer reviewed and may be re‐organized for online delivery, but are not copy‐edited or typeset. Technical support issues arising from supporting information (other than missing files) should be addressed to the authors.

Supplementary
