# Supplementary material for: Topology Effects in Molecular Organic Electronic Materials: Pyrene and Azupyrene
Source: Chemphyschem. 2021 May 7;22(11):1065–73. doi: 10.1002/cphc.202100222 (PMC8251946; doi:10.1002/cphc.202100222)
Supplement: Supplementary file 1 — Supplementary [file CPHC-22-1065-s001.pdf]

ChemPhysChem

Supporting Information

Topology Effects in Molecular Organic Electronic Materials: Pyrene and Azupyrene**

Benedikt P. Klein, Lukas Ruppenthal, Samuel J. Hall, Lars E. Sattler, Sebastian M. Weber, Jan Herritsch, Andrea Jaegermann, Reinhard J. Maurer, Gerhard Hilt,* and J. Michael Gottfried*

Supporting Information

Comparison of between Gas-Phase and Molecular Crystal Calculations

To test the influence of the molecular environment on the DFT-calculated core-electron binding energies, calculations were performed both for the gas-phase molecules and the molecular crystals of the molecules. The crystal structures were taken from X-ray diffraction experiments reported in the literature,[1] both for azupyrene and pyrene they contain four molecules per unit cell. The length of the unit cell vectors and the atomic positions were optimized in a periodic DFT calculation, while the angles of the unit cell were kept fixed to their experimental values. The optimizations were carried out using CASTEP-18.1.[2] with the PBE functional[3], a plane-wave cutoff of 500 eV and a 3×3×3 k-grid. The XPS core-level binding energies were obtained with the delta self-consistent field (ΔSCF) method of constraining electronic occupations to resemble full core-hole excitations. Figure S1 shows the results by comparing both the C1s binding energies (vertical lines) and the resulting peaks shapes. The peaks were generated by summation over the corresponding number of subpeaks for each atom. Each subpeak is represented by a pseudo-Voigt function[4] with a full width at half maximum (FWHM) of 0.7 eV and a Gaussian-to-Lorentzian ratio of 70%/30% to simulate the experimental resolution. The peaks of the free molecule (16 carbon atoms) and the molecular crystal (64 carbon atoms) were normalized to the same area and shifted to align the maxima for a better comparison of the peak shape. As can be seen, the peak of the molecular crystal of pyrene is slightly broader, but overall, the individual shifts and peak shapes are almost identical. Therefore it seems justified to compare results of the gas-phase calculations to experimental results obtained for polycrystalline thin films.

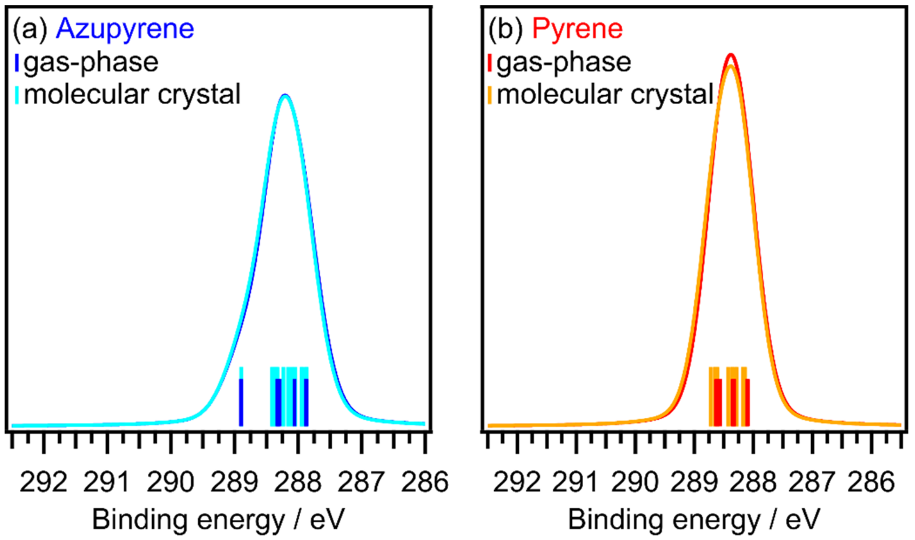

Figure S1. Comparison of the C1s XPS peak shifts and peak shapes for DFT calculations of the gas-phase molecules and molecular crystals of azupyrene (a) and pyrene (b). The core-level binding energies are displayed both as vertical lines and as combined pseudo-Voigt peaks with a FWHM of 0.7 eV and a Gaussian-to-Lorentzian ratio of 70%/30%. The energies and peaks of the free molecules were rigidly shifted to align the maxima of both peaks allowing for a better comparison of the peak shape.

MO-Projection Analysis of NEXAFS Signals

The NEXAFS simulations described in the main text can be further processed to gain insight into the origin of the observed spectral features. To achieve this aim, the NEXAFS excitations are projected onto the molecule's electronic eigenstates. The resulting final-state MO-projected excitations deconvolute the contributions with different final states to the overall spectrum. Figure S2 shows the experimental NEXAFS spectra of thin films of azupyrene (a) and pyrene (b) in comparison with the MO-projected NEXAFS simulations for the free molecules. With these simulations, the contribution of the single final state orbitals to the overall NEXAFS transitions can now be determined. The prominent first peak is for both molecules derived from the three excitations showing the transitions C1s→LUMO, LUMO+1, and LUMO+2. In the case of azupyrene, the C1s→LUMO transition is dominant, and the other transitions form an extended high energy shoulder, while for pyrene, the C1s→LUMO and C1s→LUMO+1 transitions form a double-peak structure, while the C1s→LUMO+2 transition lies beneath the C1s→LUMO+1 transition with lower intensity.

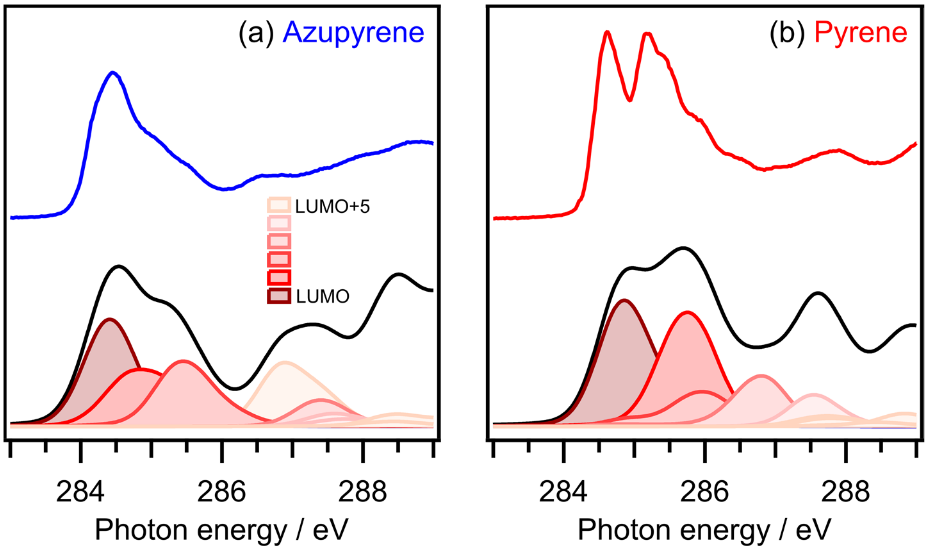

Figure S2. Comparison of MO-projected NEXAFS simulations with the experimental spectra for azupyrene (a) and pyrene (b) The first peak is in both cases consisting out of the C1s→LUMO, LUMO+1, and LUMO+2 excitations with the C1s→LUMO excitation forming the leading edge. The calculated spectra were rigidly shifted by 6.2 eV to match the experimental energy scale. The calculated spectra were already published in the context of method development.[5]

Dipole-Forbidden Transitions in Azupyrene

While the HOMO→LUMO (S_0→S_1) transitions of azupyrene is dipole forbidden by symmetry selection rules, it may still be visible as a weak absorption band in the UV/Vis spectrum. Figure S3 shows a logarithmic plot of the UV/Vis absorption spectrum, which allows for a better inspection of weak features. The azupyrene solution shows a broad absorption band at lower energy than the main absorption attributed to the HOMO→LUMO+1 (S_0→S_3) transition at 480 nm (2.5 eV). The broad band in the range of 600 to 700 nm (2.0 to 1.7 eV) is very weak compared to the other features in the spectrum, but the intensity is still significantly higher than in the same range of the pyrene spectrum. See Tables S1 and S2 for a complete description of all transitions and Figure S5 for a full Jablonski diagram.

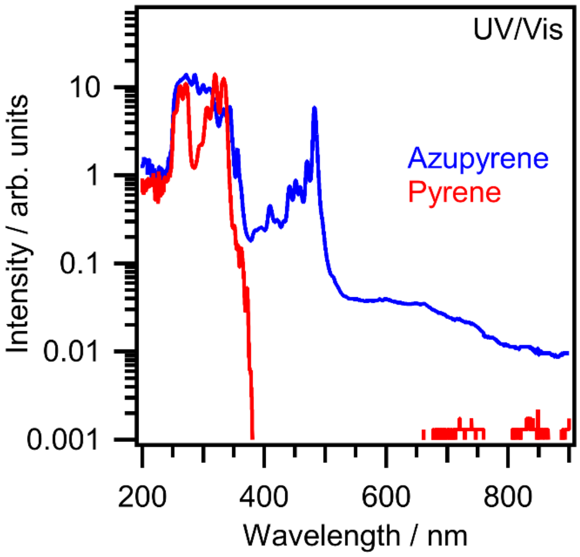

Figure S3. UV/Vis spectra of azupyrene (blue) and pyrene (red). The depicted spectra are the same as shown in Figure 3a of the main text, but with a logarithmic intensity scale.

Fluorescence Spectra and Full Jablonski Scheme

The fluorescence spectra depicted in Figure S4 were recorded using a *Cary Eclipse* fluorescence spectrophotometer with an excitation wavelength of 270 nm. The measurements were performed with solutions of the molecules in cyclohexane, the concentration was 1 mmol·l^{-1}. For pyrene, the broad fluorescence peak at 400 nm, known from the literature, is visible.[6] Around 800 nm the second order satellite from the same transition can be detected. For azupyrene, no fluorescence can be seen.

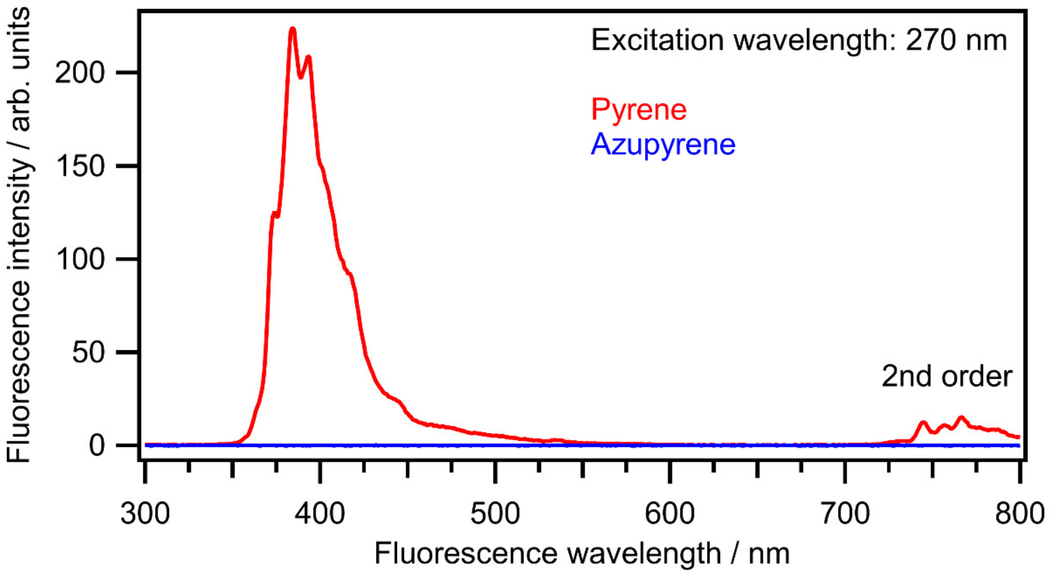

Figure S4. Fluorescence spectra of azupyrene (blue) and pyrene (red). While the pyrene spectrum shows the fluorescence peak well known in literature,[6] no fluorescence was detected for azupyrene.

This lack of fluorescence for the azupyrene molecules can be explained by the symmetry and energetic position of its electronic states as depicted in the Jablonski diagram (Figure S5). The energies and oscillator strengths used in Figure S5 are compiled in Table S1 and S2 together with the electron configurations of the excited states S_n.

For pyrene, the $S_0 \rightarrow S_1$ (HOMO→LUMO) transition is dominating in the absorption spectrum as well as in the fluorescence spectrum. For azupyrene, the transitions $S_0 \rightarrow S_1$ and $S_0 \rightarrow S_2$ are dipole-forbidden due to the symmetry of the involved orbitals. However, the $S_0 \rightarrow S_3$ (HOMO→LUMO+1) transition of azupyrene is still at relatively low energy, leading to an absorption peak in the visible range. According to Kasha's rule, the excited molecules relax by non-radiative means to the lowest lying singlet state S_1. For pyrene, a fluorescence transition from $S_1 \rightarrow S_0$ follows. For azupyrene, however, this transition is dipole-forbidden, leading to the absence of fluorescence and a non-radiative return to the ground state.

We found no indications of fluorescence from higher states or phosphorescence decay. Fluorescence from a higher electronic state, in this case S_3-fluorescence, requires a very large energy difference between the excited states, which is not fulfilled for azupyrene. A further

factor, which could contribute to the non-radiative decay possibilities in azupyrene, is the Stone-Wales rearrangement, which may be mediated by the excited S_1 state.[7]

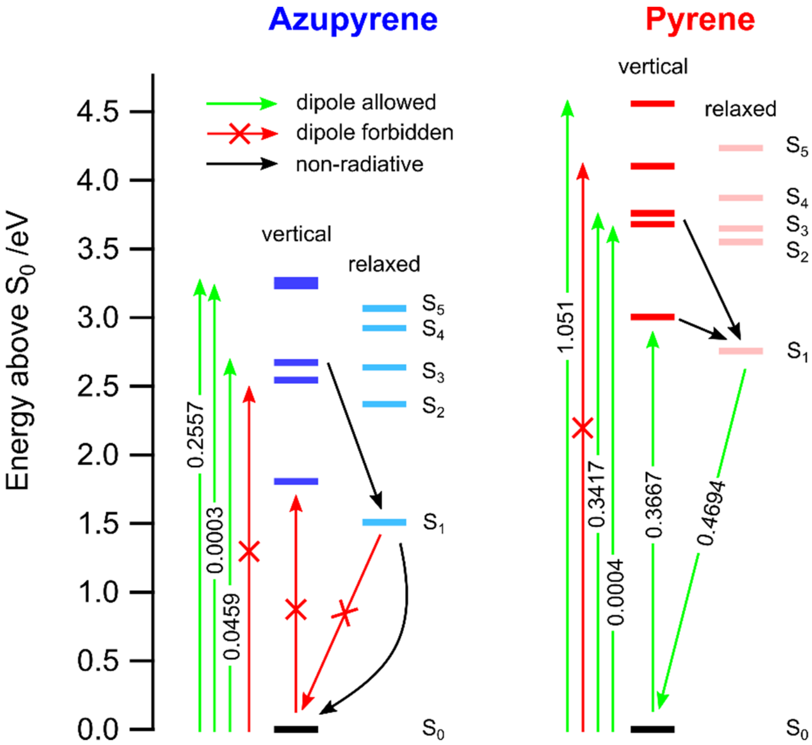

Figure S5. Jablonski diagrams of azupyrene and pyrene. The energies of the vertical S_n states were calculated for the S_0 molecular structure, while the energies for the relaxed S_n states were calculated after a structural optimization with the excited electron configuration. All energies were obtained with DFT using the B3LYP functional. The oscillator strengths for each transition were calculated with the CC2 method using the corresponding DFT-optimized structure. Electron configurations for the excited states, oscillator strengths and all energies are compiled in Tables S1 and S2, the basis set was def2-TZVPP.

Table S1. Electron configurations and energies of the excited states for **azupyrene**. Vertical and relaxed state energies were obtained with the B3LYP functional. The transition energies and oscillator strengths for each transition were calculated with the CC2 method using the corresponding DFT-optimized structure. All energies are in eV, the oscillator strengths are dimensionless, the basis set was def2-TZVPP.

	**Electron configuration change from ground state S_0 to excited state S_n**	**vertical energy difference of S_n relative to S_0**	**relaxed energy difference of S_n relative to S_0**	**transition energy from S_0 to S_n**	**oscillator strength**
S_5	$(HOMO)^{-1}$ $(LUMO+1)^{+1}$	3.27	3.07	4.21	0.2557
S_4	$(HOMO)^{-1}$ $(LUMO+2)^{+1}$	3.23	2.92	3.82	0.0003
S_3	$(HOMO)^{-1}$ $(LUMO+1)^{+1}$	2.67	2.64	2.95	0.0459
S_2	$(HOMO-1)^{-1}$ $(LUMO)^{+1}$	2.54	2.37	2.93	0
S_1	$(HOMO)^{-1}$ $(LUMO)^{+1}$	1.81	1.51	1.95	0

Table S2. Electron configurations and energies of the excited states for **pyrene**. Vertical and relaxed state energies were obtained with the B3LYP functional. The transition energies and oscillator strengths for each transition were calculated with the CC2 method using the corresponding DFT-optimized structure. All energies are in eV, the oscillator strengths are dimensionless, the basis set was def2-TZVPP.

	**Electron configuration change from ground state S_0 to excited state S_n**	**vertical energy difference of S_n relative to S_0**	**relaxed energy difference of S_n relative to S_0**	**transition energy from S_0 to S_n**	**oscillator strength**
S_5	$(HOMO)^{-1}$ $(LUMO+1)^{+1}$	4.56	4.24	5.64	1.0510
S_4	$(HOMO)^{-1}$ $(LUMO+2)^{+1}$	4.10	3.87	4.57	0
S_3	$(HOMO-1)^{-1}$ $(LUMO)^{+1}$	3.76	3.65	4.84	0.3417
S_2	$(HOMO)^{-1}$ $(LUMO+1)^{+1}$	3.68	3.55	3.75	0.0004
S_1	$(HOMO)^{-1}$ $(LUMO)^{+1}$	3.00	2.76	4.05	0.3667

NMR Spectra

The NMR spectra, from which the shifts discussed in the main text were derived, are depicted in Figure S6. The theoretical peak positions in the lower part calculated were calculated according to the continuous set of gauge transformations (CSGT) method[8] on with the B3LYP functional and the def2-TZVPP basis set. The calculated peak positions were furthermore shifted, such that the weighted average of peaks was the same for theory and experiment.

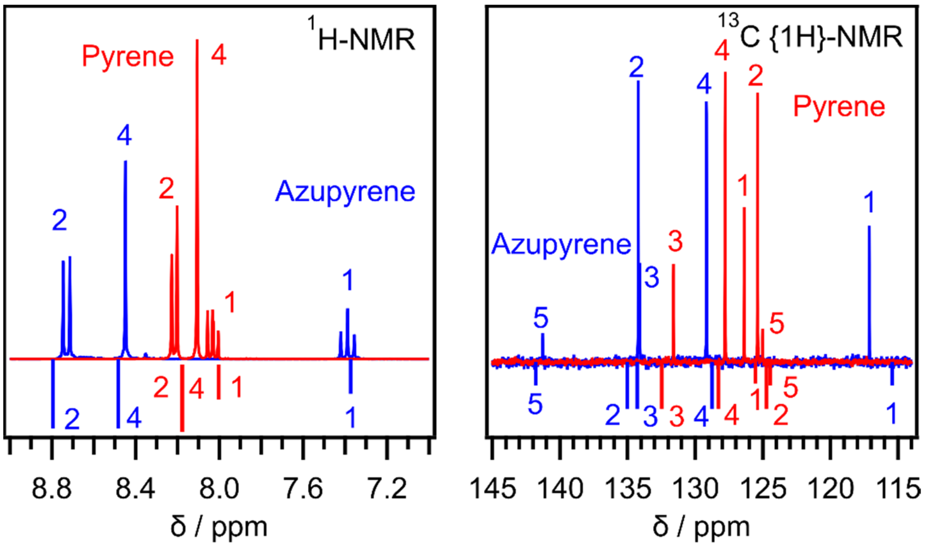

Figure S6. ^{1}H (left) and ^{13}C (right) NMR spectra of azupyrene (blue) and pyrene (red). The upper part shows the experimental spectra, below them, vertical lines represent the peak positions calculated by DFT (B3LYP/def2-TZVPP, CSGT calculations). The theoretical spectra were shifted, such that the weighted average of all peaks was the same for theory and experiment.

NICS analysis

In addition to the HOMA approach laid out in the main text, we also aimed to quantify the aromaticity in pyrene and azupyrene in a spatially resolved fashion with the nucleus-independent chemical shift (NICS) method.[9] This method is based on the computed aromatic ring current induced by a (hypothetical) external magnetic field perpendicular to the plane of the aromatic system. Negative NICS values (diatropic ring current) indicate aromaticity, while antiaromaticity is characterized by positive values (paratropic ring current).

In the following, we discuss the so called NICS(0) values. The "(0)" indicates that the NICS value was calculated for a point in the molecular plane, in contrast to the alternative NICS(1) value, which would be calculated for a point 1 Å above the molecular plane. All calculations were done on the PBE with the def2-TZVPP basis set.

To gain some similar systems for comparison, we did not only calculate the NICS values for pyrene and azupyrene, but also for benzene, naphthalene and azulene. The results are compiled in Table S3. The NICS values were calculated for the center point of each symmetry independent ring in each of the molecules. All values are negative, unsurprisingly indicating aromaticity for all molecules.

The values for the pyrene show a higher grade of aromaticity for the apical rings compared to the lateral rings, in agreement with the HOMA analysis and the model of a doubly ethenediyl bridged biphenyl with two Clar sextets (see Figure 7 of the main text).

Azupyrene shows a higher grade of aromaticity for the 5-membered rings compared to the 7-membered rings. However, this is mainly due to the dependence of the NICS values on the ring size, as can be seen when comparing the azulene and the naphthalene molecules. In the azulene molecule, the 5-membered ring again has a much larger (more negative) NICS value compared to the 7-membered ring.

Table S3. NICS(0) values for benzene, naphthalene, azulene, pyrene and azupyrene. The calculations were performed with the PBE functional, the basis set was def2-TZVPP.

Molecule	Ring size/position	NICS(0) value / ppm
Benzene	6-membered	-7.9
Naphthalene	6-membered	-8.3
Azulene	5-membered	-16.6
	7-membered	-5.2
Pyrene	6-membered, apical	-10.9
	6-membered, lateral	-3.6
Azupyrene	5-membered	-18.4
	7-membered	-4.3

Synthesis of Azupyrene

General Information

All reactions with water- and/or air-sensitive starting materials were carried out in pre-dried glass wares under Argon atmosphere utilizing standard Schlenk techniques. All used solvents were dried over molecular sieves (3 Å) and were degassed prior to use. Thin layer chromatography (TLC) was carried out on prefabricated plates (silica gel 60, F254 with fluorescence indicator) by *Macherey Nagel*. Column Chromatography was carried out on silica gel 60 (40-63 µm, 230-400 mesh) by *Macherey Nagel*. Commercially available chemicals were used without further purification. Non-commercial reagents were synthesized by literature-known procedures.

^{1}H and ^{13}C NMR were either recorded on a *Bruker Fourier 300HD* or a *Bruker Avance III 500HD* spectrometer at room temperature utilizing preset pulse programs. The chemical shifts are given in parts per million (ppm). The residual solvent signal ($CDCl_3$: ^{1}H NMR: δ = 7.26 ppm, ^{13}C NMR: δ = 77.16 ppm, DMSO-d_6: ^{1}H NMR: δ = 2.50 ppm, ^{13}C NMR: δ = 39.52 ppm, CD_3CN: ^{1}H NMR: δ = 1.94 ppm, ^{13}C NMR: δ = 118.26 ppm) was used for calibration referred to tetramethylsilane. Infrared spectra were recorded on a *Shimadzu IRSpirit* FT-IR spectrometer. The absorption bands are given in wave numbers (cm^{-1}). High resolution mass spectra by electron spray ionization (ESI) were recorded on a *Waters Q-TOF Premier* spectrometer. The ionization was accomplished with an energy of 3 kV. High resolution mass spectra by electron impact ionization (EI) were recorded on a *Thermo Scientific DFS* spectrometer with an ionization energy of 70 eV. GC/MS spectra were recorded on a *Shimadzu GC 2010 Plus* gas chromatograph coupled with a *Shimadzu QP2020* mass detector, using electron impact ionization (EI) at an energy of 70 eV.

Reaction Sequence

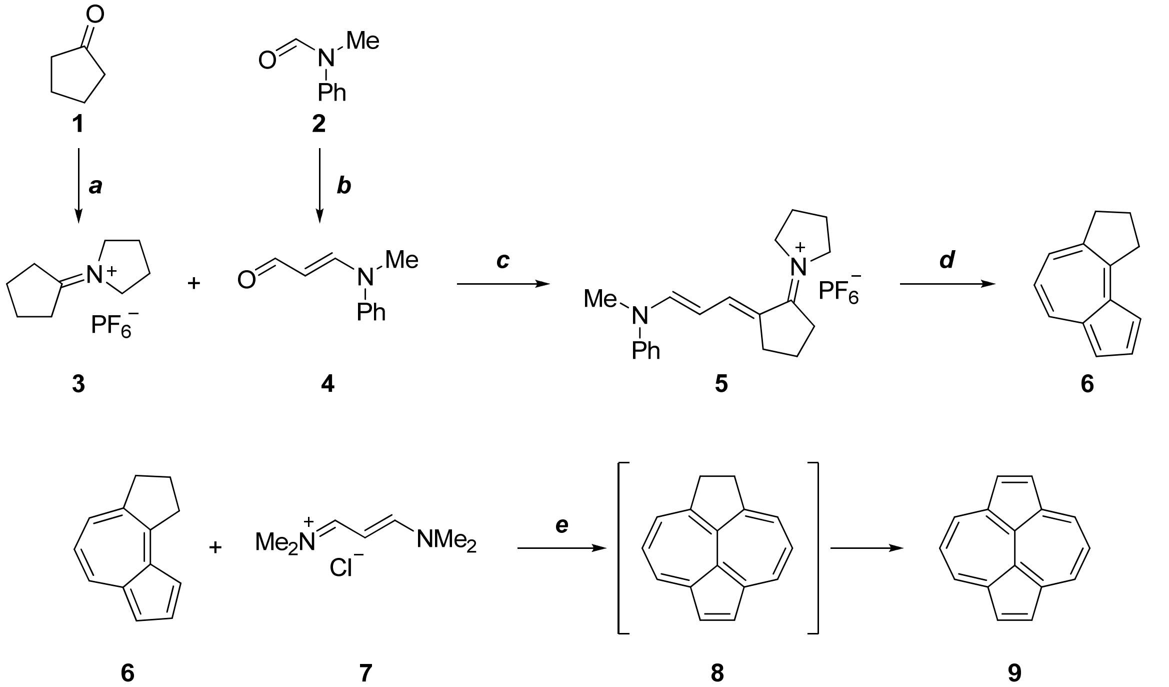

Scheme S1: Schematic overview of the azupyrene synthesis sequence. a) Cyclopentanone (1.0 equiv.), pyrrolidine (1.2 equiv.), ammonium hexafluorophosphate (1.0 equiv.) in toluene, 135 °C, Dean-Stark trap; b) *N*-methylformanilid (1.0 equiv.), butyl vinyl ether (1.1 equiv.), oxalyl chloride (1.1 equiv.) in acetonitrile, -10 °C; c) 1-cyclopentylidenepyrrolidinium hexafluorophosphate (1.0 equiv.), (*E*)-3-(methyl(phenyl)amino)acrylaldehyde (1.0 equiv.), acetic anhydride (1.3 equiv.), pyridine (0.4 equiv.) in CH_2Cl_2, ambient temperature; d) 1-((*E*)-2-((*E*)-3-(methyl(phenyl)amino)allylidene)cyclopentylidene)pyrrolidinium hexafluorophosphate (1.0 equiv.), sodium cyclopentadienylide (1.0 equiv.) in pyridine, sealed tube, 40 °C for 12 h, then 120 °C for 24 h; e) azulene (1.0 equiv.), (*E*)-*N*-(3-(dimethylamino)allylidene)-*N*-methylmethanaminium chloride (1.5 equiv.), sodium methanolate (10 equiv.) in propylene carbonate, first ambient temperature for 5 h, then 90 °C / 12 h, 150 °C / 2 h and finally 200 °C over 3 d.

Experimental Section

1-Cyclopentylidenepyrrolidinium hexafluorophosphate (3)

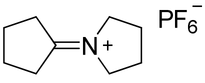

3

According to a literature-known procedure by Saba,[10] cyclopentanone (2.3 mL, 29.0 mmol, 1.45 equiv.), pyrrolidine (2.2 mL, 35.3 mmol, 1.76 equiv.) and ammonium hexafluorophosphate (3.26 g, 20 mmol, 1.0 equiv.) were suspended in toluene (40.0 mL). The reaction mixture was heated utilizing a Dean-Stark trap to 135 °C for 2 h. After that, the mixture was cooled to 0 °C whereby the product crystallized. The solid was filtered and washed with Et_2O (20.0 mL) twice. The product **3** (5.36 g, 18.9 mmol, 95%) was obtained as a beige solid and it could be used without further purifications.

^{1}H NMR (500 MHz, DMSO-d_6) δ = 3.83 (ddq, *J* = 7.1, 4.7, 2.3 Hz, 2H), 2.79 (qt, *J* = 4.7, 2.2 Hz, 2H), 2.09 – 2.00 (m, 2H), 1.97 – 1.86 (m, 2H) ppm.

The analytical data are in accordance with the literature.[10]

3-(Methyl(phenyl)amino)acrylaldehyde (4)

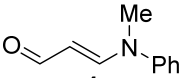

4

According to a literature-known procedure by Hayashi,[11] oxalyl chloride (4.7 mL, 55.1 mmol, 1.10 equiv.) was dissolved in acetonitrile (10 mL) and the mixture was cooled to −10 °C. Then a mixture consisting of *N*-methylformanilide (6.2 mL, 50.1 mmol, 1.0 equiv.) and butyl vinyl ether (6.9 mL, 53.6 mmol, 1.07 equiv.) in 10.0 mL acetonitrile was added dropwise to the solution over a period of 30 min. The reaction mixture may not warm up over −5 °C during the addition. After the addition, the reaction mixture was allowed to warm to ambient temperature, and it was stirred for an additional hour. After that, the mixture was cooled to 0 °C and saturated sodium carbonate (30 mL) was slowly added. Toluene was added and the aqueous phase was extracted with toluene three times. The organic phase was washed with brine, dried over $MgSO_4$, filtered and the solvent was removed under reduced pressure. The crude product was crystallized from isopropanol/*n*-pentane at 0 °C. The product **4** (4.93g, 30.5 mmol, 61%) was isolated as a beige solid.

^{1}H NMR (300 MHz, $CDCl_3$) δ = 9.26 (d, *J* = 8.1 Hz, 1H), 7.52 (d, *J* = 13.1 Hz, 1H), 7.39 (dd, *J* = 8.5, 7.3 Hz, 2H), 7.25 – 7.12 (m, 3H), 5.44 (dd, *J* = 13.1, 8.1 Hz, 1H), 3.31 (s, 3H) ppm.

^{13}C NMR (126 MHz, $CDCl_3$) δ = 190.3, 146.2, 129.8 (2C), 125.5, 120.7, 105.8, 31.0 ppm.

The analytical data are in accordance with the literature.[11]

1-(-2-(3-(Methyl(phenyl)amino)allylidene)cyclopentylidene)pyrrolidinium hexafluorophosphate (5)

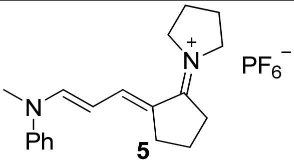

5

In accordance to a literature-known procedure by Jutz,[12] 1-cyclopentylidenepyrrolidinium hexafluorophosphate (2.54 g, 8.97 mmol, 1.00 equiv.) was dissolved in 6.0 mL CH_2Cl_2 giving a red solution. Afterwards, 3-(methyl(phenyl)amino)acrylaldehyde (1.47 g, 9.12 mmol, 1.02 equiv.), acetic anhydride (1.1 mL, 11.6 mmol, 1.29 equiv.) and pyridine (0.3 mL, 3.72 mmol, 0.41 equiv.) were added to the solution resulting in a dark red mixture and stirred overnight. The product was precipitated by the addition of Et_2O. The resulting suspension was filtered, and the obtained crude product was purified by dissolving in CH_2Cl_2 again followed by precipitation with Et_2O. This procedure was repeated twice. The product **5** was obtained after drying *in vacuo* (3.02 g, 7.08 mmol, 79%) as a brown solid.

^{1}H NMR (300 MHz, CD_3CN) δ = 7.79 (d, *J* = 12.3 Hz, 1H), 7.67 (d, *J* = 11.8 Hz, 1H), 7.50 – 7.38 (m, 2H), 7.37 – 7.22 (m, 3H), 5.63 (t, *J* = 12.0 Hz, 1H), 3.84 (t, *J* = 6.6 Hz, 2H), 3.81 – 3.70 (m, 4H), 3.43 (s, 3H), 2.83 (t, *J* = 7.6 Hz, 2H), 2.76 – 2.66 (m, 4H), 2.18 – 2.06 (m, 5H), 2.04 – 1.90 (m, 8H) ppm.

^{13}C NMR (75 MHz, CD_3CN) δ = 175.6, 150.2, 130.6, 126.7, 121.6, 104.1, 56.1, 55.8, 54.8, 38.3, 36.8, 31.9, 26.5, 25.3, 25.3, 24.7, 21.7 ppm.
IR (ATR) $\tilde{\nu}$ = 2986, 2890, 1709, 1622, 1583, 1579, 1533, 1530, 1493, 1457, 1449, 1436, 1401, 1360, 1340, 1326, 1300, 1276, 1250, 1209, 1193, 1164, 1124, 1102, 1031, 974, 961, 879, 830, 791, 776, 761, 741, 693, 643, 556, 526, 501 cm^{-1}.
HRMS (ESI$^+$): *m/z* [M]$^+$ calcd for $C_{19}H_{25}N_2^+$: 281.2012, found: 281.2013, HRMS (ESI$^-$): *m/z* [A]$^-$ calcd. for PF_6^-: 144.9647, found 144.9639.
Note: The ^{1}H NMR spectra shows impurities due to 1-cyclopentylidenepyrrolidinium hexafluorophosphate, which results in inconsistent integral ratios in the range from 4 to 1.5 ppm.

2,3-Dihydro-1*H*-cyclopenta[e]azulene (6)

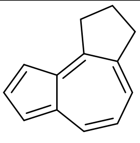
6

In accordance to a literature-known procedure by Jutz,[12] 1-((*E*)-2-((*E*)-3-(methyl(phenyl)amino)allylidene)cyclopentylidene)pyrrolidinium hexafluorophosphate (1.28 g, 3.00 mmol, 1.00 equiv.) was dissolved in 6.0 mL pyridine in a sealed tube. Afterwards, 1.55 mL (3.10 mmol, 1.03 equiv.) of sodium cyclopentadiene solution (2.0 M in tetrahydrofuran) was added dropwise. The resulting mixture was stirred at 40 °C for 12 h and heated up to 120 °C for additional 24 h. The solvent was removed *in vacuo*. The residue was dissolved in CH_2Cl_2 and filtered through a short pad of silica. The filtrate was washed with 60 mL of 1 M hydrochloric acid. The organic layer was dried over $MgSO_4$, filtered and the solvent was removed under reduced pressure. The product **6** was obtained after column chromatography (eluent: *n*-pentane) as a blue oil (65 mg, 0.386 mmol, 13%).
^{1}H NMR (300 MHz, $CDCl_3$) δ = 8.29 (d, *J* = 9.5 Hz, 1H), 7.85 (t, *J* = 3.8 Hz, 1H), 7.65 (d, *J* = 10.0 Hz, 1H), 7.30 (d, *J* = 3.0 Hz, 1H), 7.24 (d, *J* = 3.7 Hz, 1H), 7.10 (t, *J* = 9.8 Hz, 1H), 3.56 (t, *J* = 7.6 Hz, 2H), 3.28 (t, *J* = 7.4 Hz, 2H), 2.22 (pent, *J* = 7.5 Hz, 2H) ppm.
GC/MS (EI+): *m/z* = 169 (13), 168 ([M]$^+$, 100), 167 (68), 166 (13), 165 (40), 164 (6), 154 (2), 153 (21), 152 (36), 139 (7), 83 (11), 82 (11), 63 (5).
The analytical data are in accordance with the literature.[13]

***N*-(3-(Dimethylamino)allylidene)-*N*-methylmethanaminium chloride (7)**

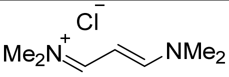

7

According to a modified procedure described by Anderson,[13] dimethylamine hydrochloride (1.63 g, 20.0 mmol, 1.0 equiv.) was suspended in ethanol (14.0 mL). Then dimethylaminoacroleine (2.0 mL, 20.0 mmol, 1.0 equiv.) was added and the suspension was stirred at 80 °C for 2 days. After that, the reaction mixture was cooled to ambient temperature and the solvent was removed *in vacuo*. The residue was washed twice with cold Et_2O. The obtained crude product was recrystallized from acetone. The product **7** (2.26 g, 13.9 mmol; 69%) was isolated as a beige solid.
^{1}H NMR (300 MHz, $CDCl_3$) δ = 8.52 (d, *J* = 11.5 Hz, 2H), 5.10 (t, *J* = 11.5 Hz, 1H), 3.27 (s, 6H), 3.01 (s, 6H) ppm.
^{13}C NMR (126 MHz, $CDCl_3$) δ = 164.6 (2C), 90.0, 46.3, 38.3 ppm.
The analytical data is in accordance with the literature.[13] The product is moisture sensitive and decomposes under non-inert gas conditions.

Dicyclopenta[ef,kl]heptalene (Azupyrene, 9)

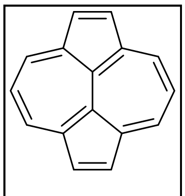
9

Following a modified procedure by Anderson,[13] azulene **6** (168 mg, 1.00 mmol, 1.0 equiv.) was dissolved in propylene carbonate (15 mL) and imine **7** (244 mg, 1.50 mmol, 1.5 equiv.) were added to the solution. Then sodium methanolate (540 mg, 10.0 mmol, 10 equiv.), dissolved in 5 mL methanol (2.5 M) were added. The mixture was stirred at room temperature for 5 h. After that, the reaction mixture was slowly heated to 90 °C for 12 h. Then the reaction mixture was heated to 150 °C for 2 h and at last it was heated to 200 °C and stirred for 3 d. The solvent was removed *in vacuo* and the residue was dissolved in

CH_2Cl_2 and filtered over Al_2O_3. The filtrate was washed with 1 M hydrochloric acid and distilled H_2O. The organic layer was dried over $MgSO_4$, filtered and the solvent was removed under reduced pressure. The product **9** was obtained after column chromatography (eluent: *n*-pentane/toluene 4:1) as a light brown solid (45.0 mg, 0.22 mmol, 22%).
^{1}H NMR (500 MHz, $CDCl_3$) δ = 8.71 (d, *J* = 9.6 Hz, 4H), 8.43 (s, 4H), 7.37 (t, *J* = 9.6 Hz, 2H) ppm.
^{13}C NMR (126 MHz, $CDCl_3$) δ = 141.0 (2C), 133.9 (4C), 133.8 (4C), 128.9 (4C), 116.8 (2C) ppm.
HRMS(EI+): *m/z* $[M]^+$ calcd for $C_{16}H_{10}$: 202.0777, found: 202.07776.

NMR Spectra

1-Cyclopentylidenepyrrolidinium hexafluorophosphate

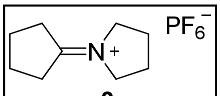

3

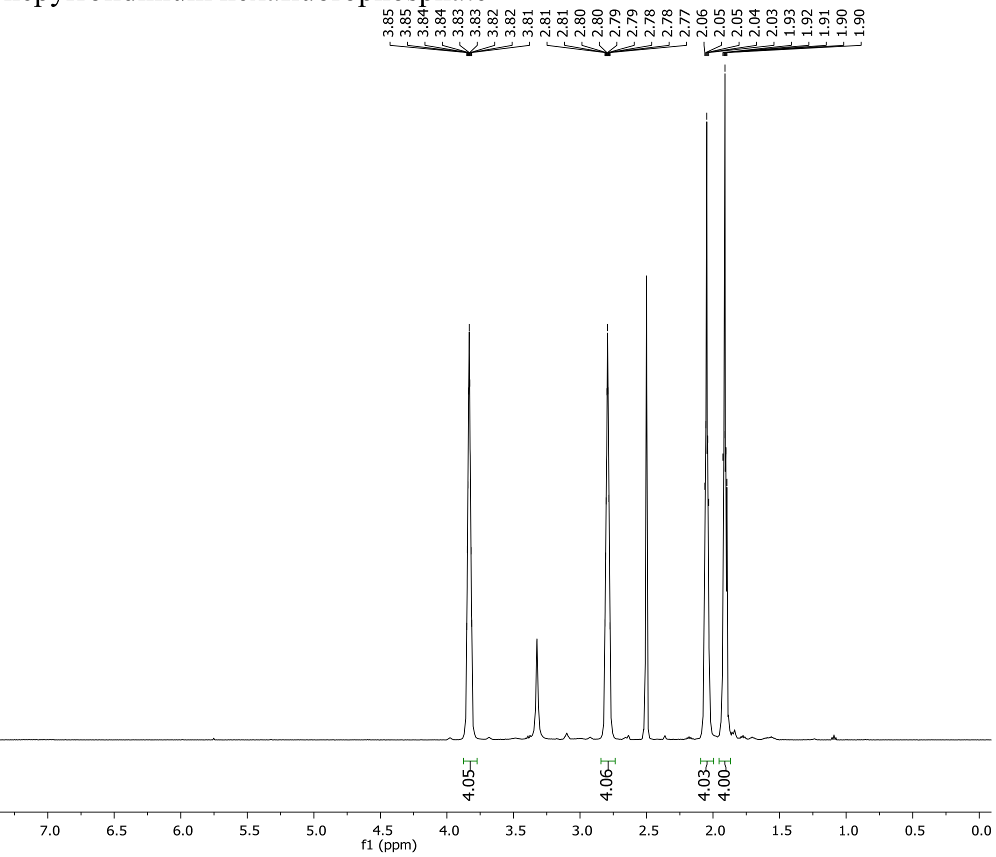

3-(Methyl(phenyl)amino)acrylaldehyde

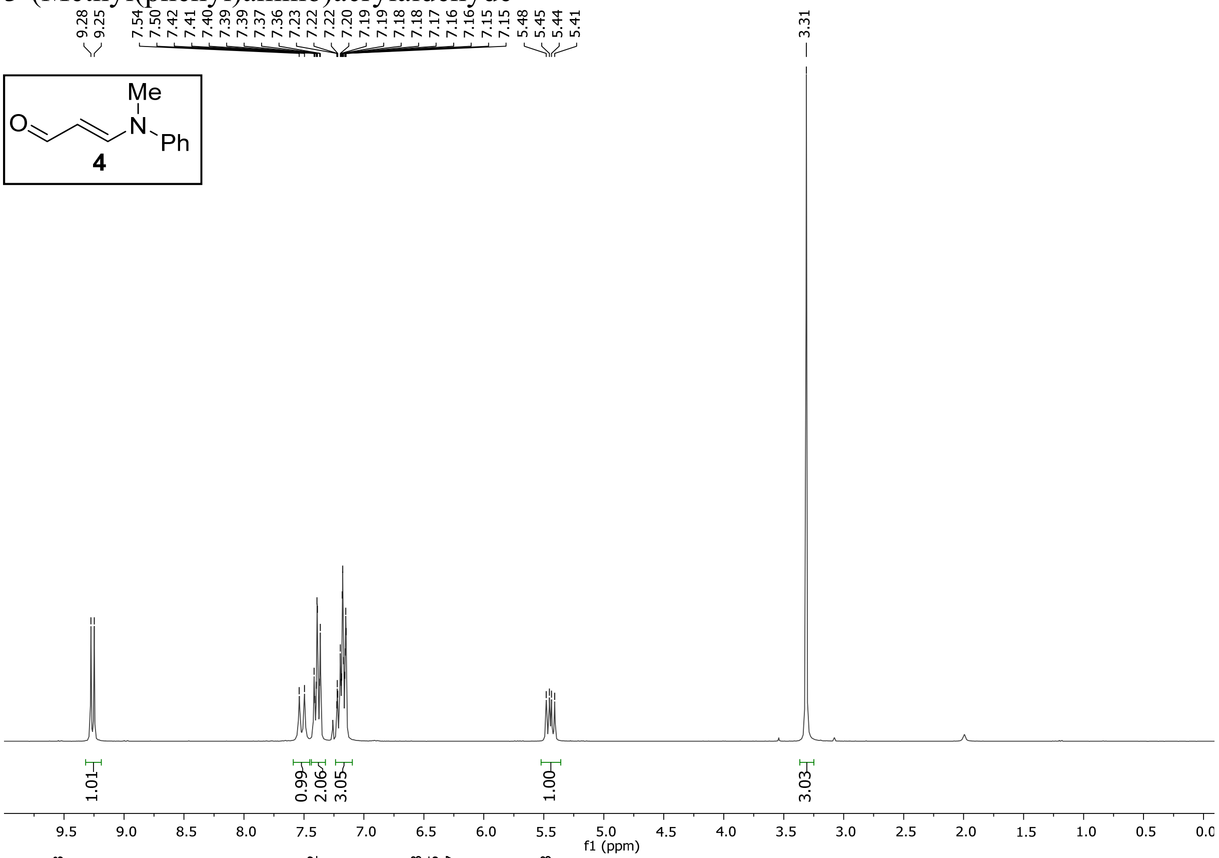

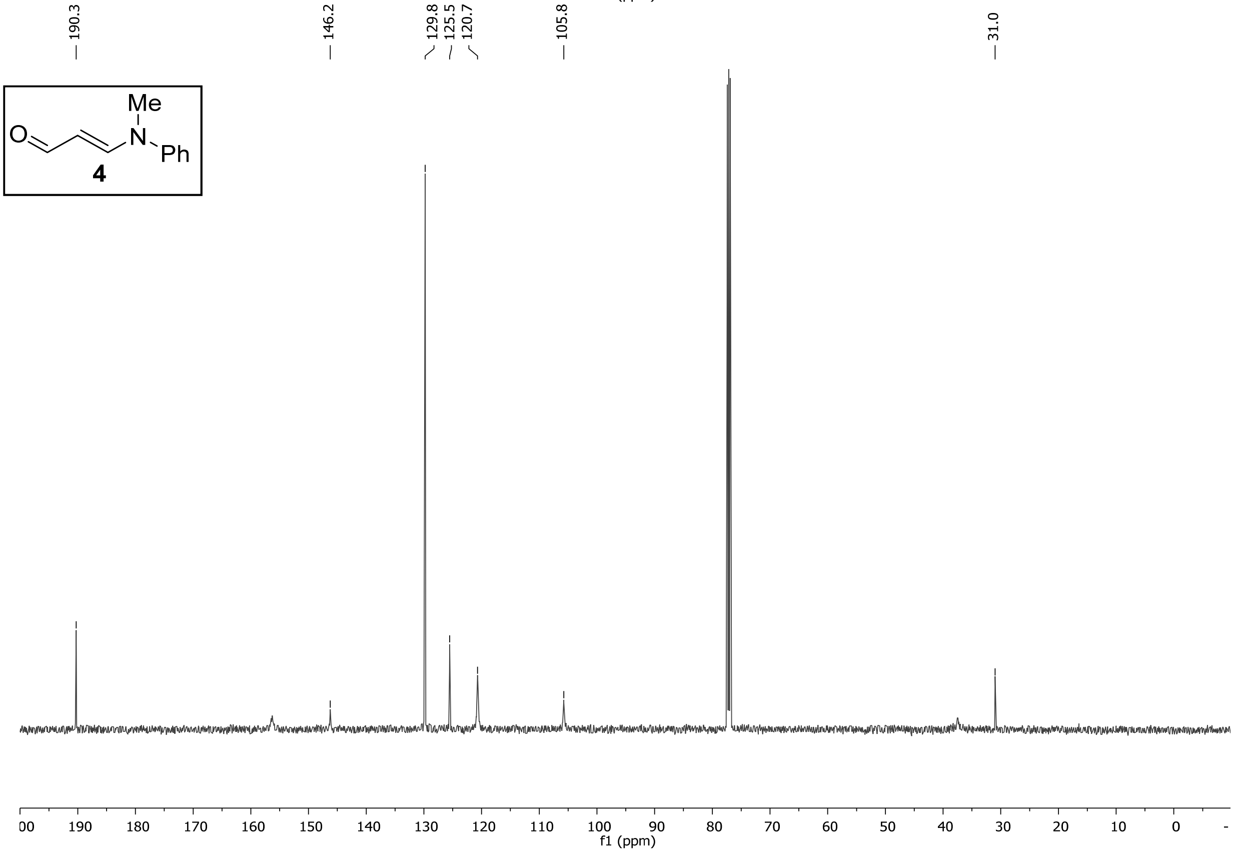

1-(2-(3-(Methyl(phenyl)amino)allylidene)cyclopentylidene)pyrrolidinium hexafluorophosphate

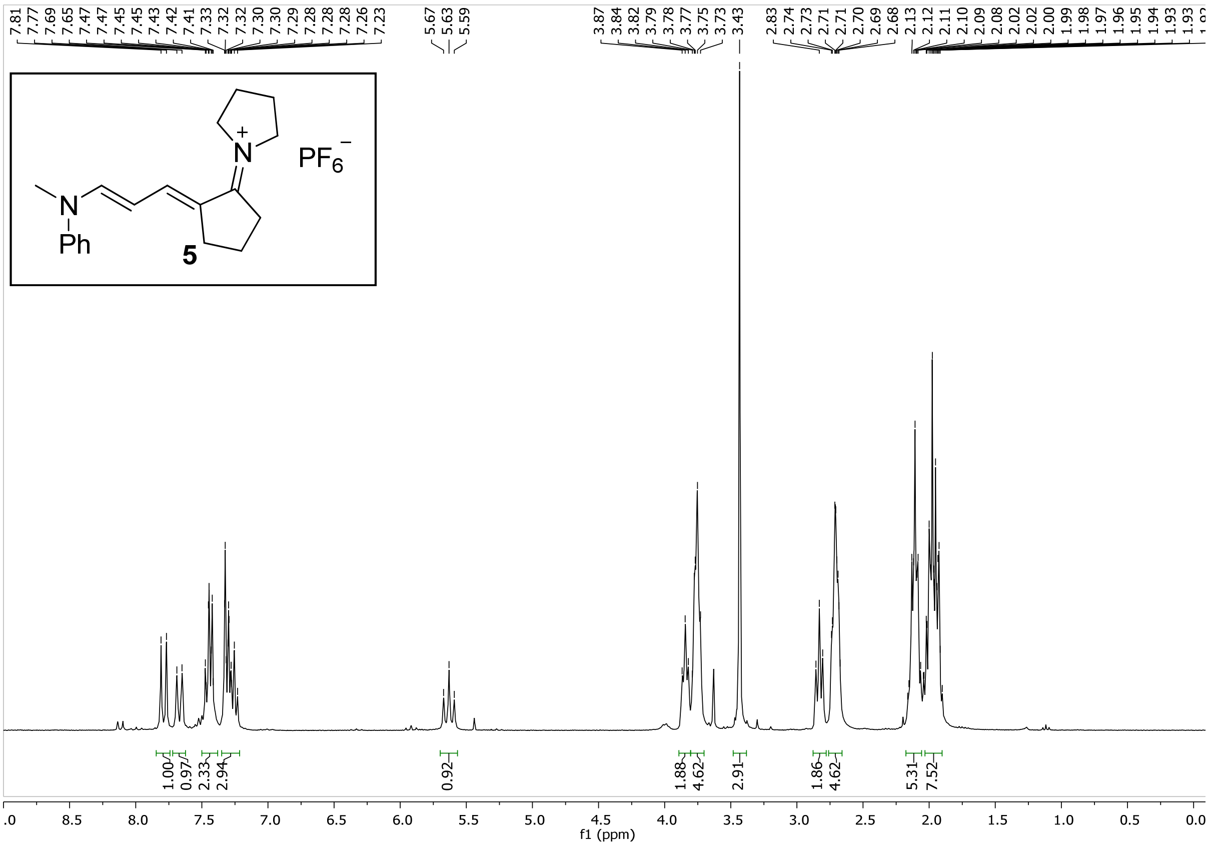

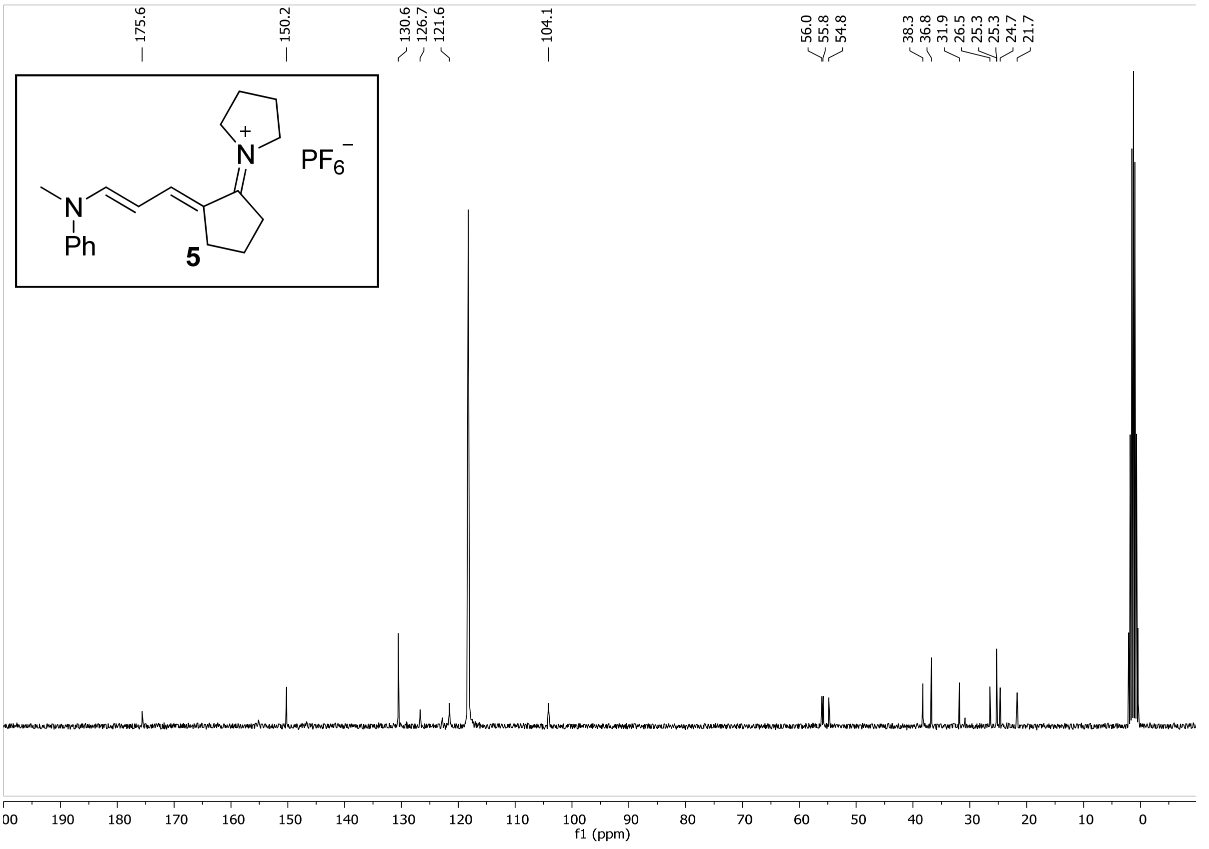

2,3-Dihydro-1*H*-cyclopenta[e]azulene

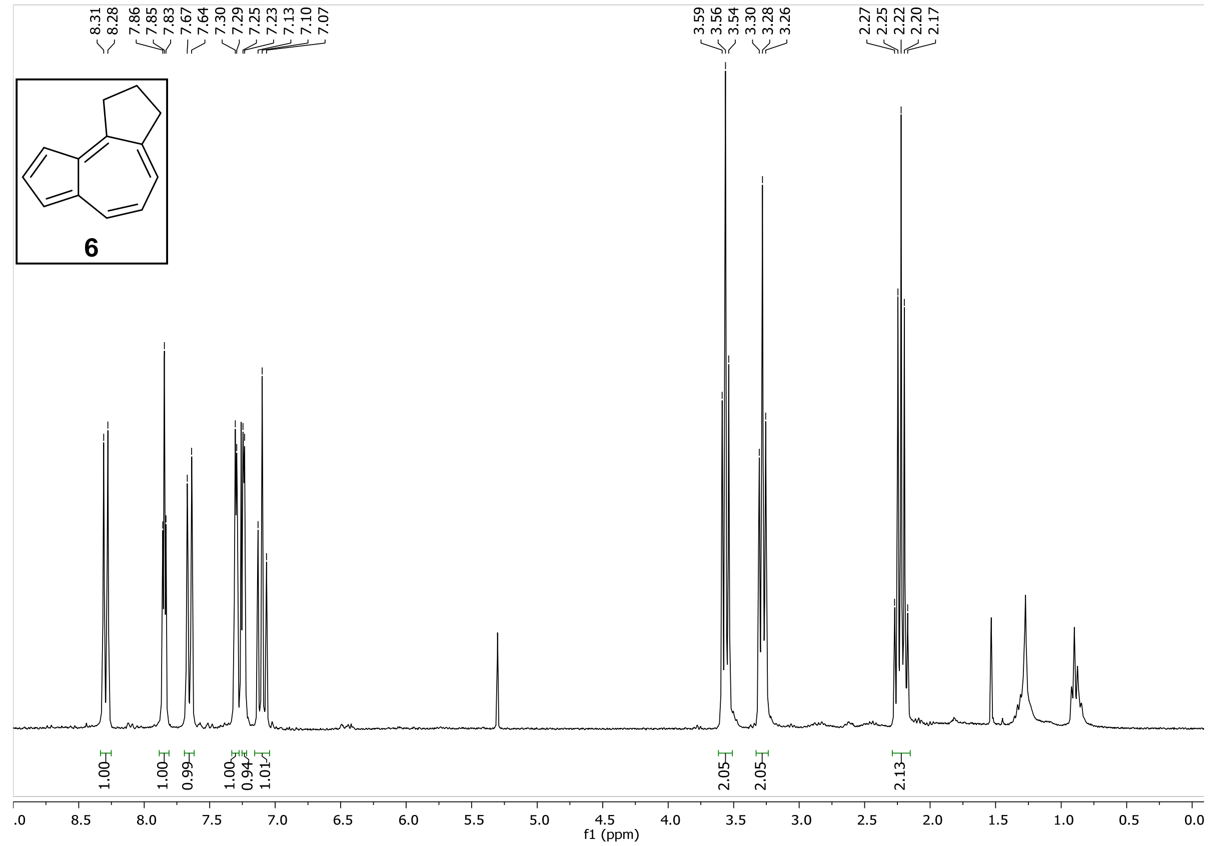

N-(3-(Dimethylamino)allylidene)-*N*-methylmethanaminium chloride

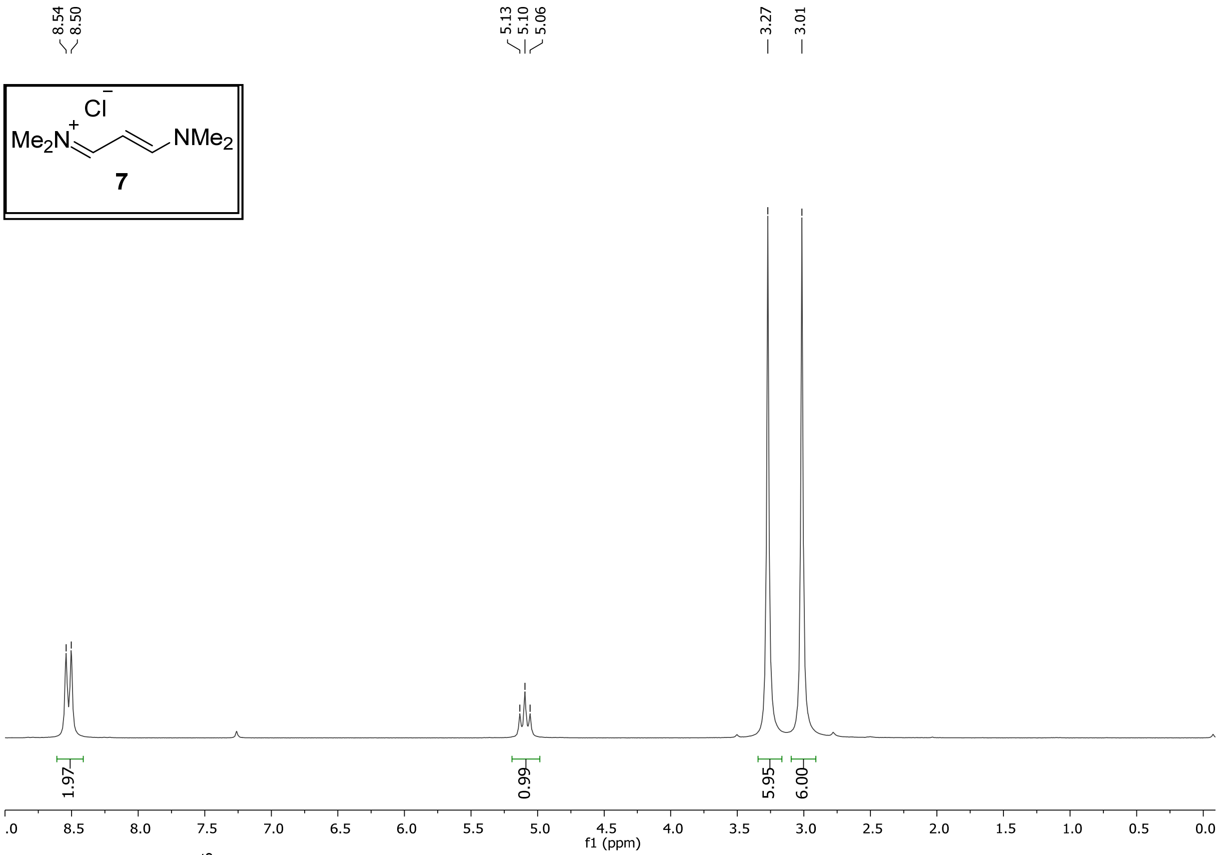

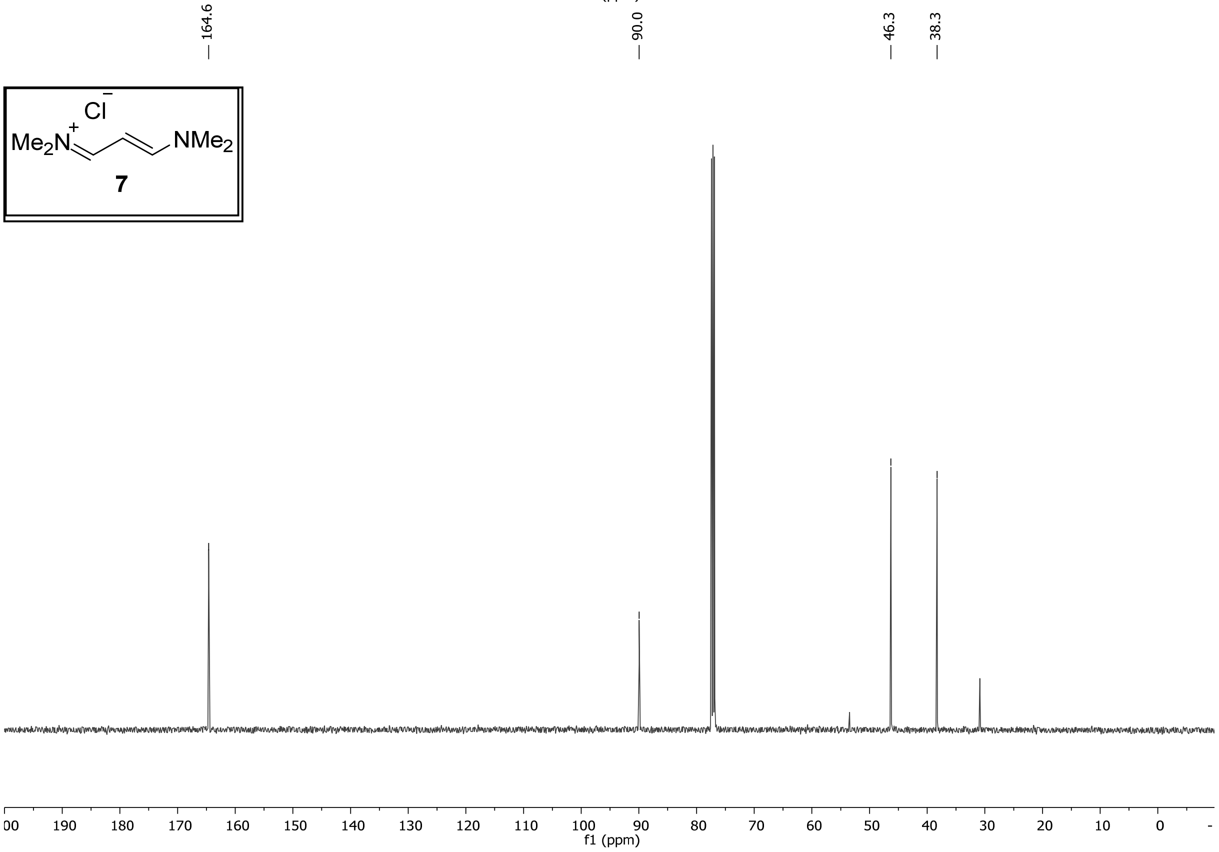

Dicyclopenta[ef,kl]heptalene (Azupyrene)

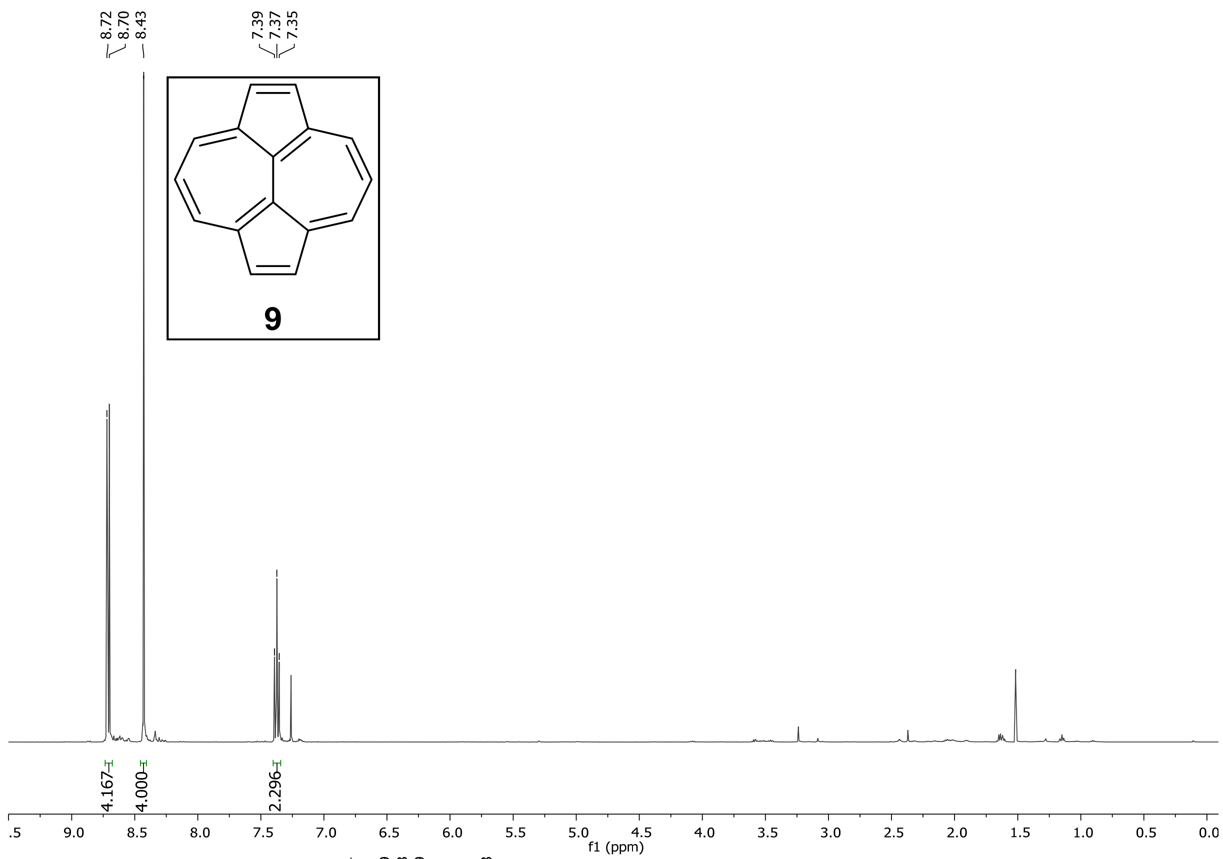

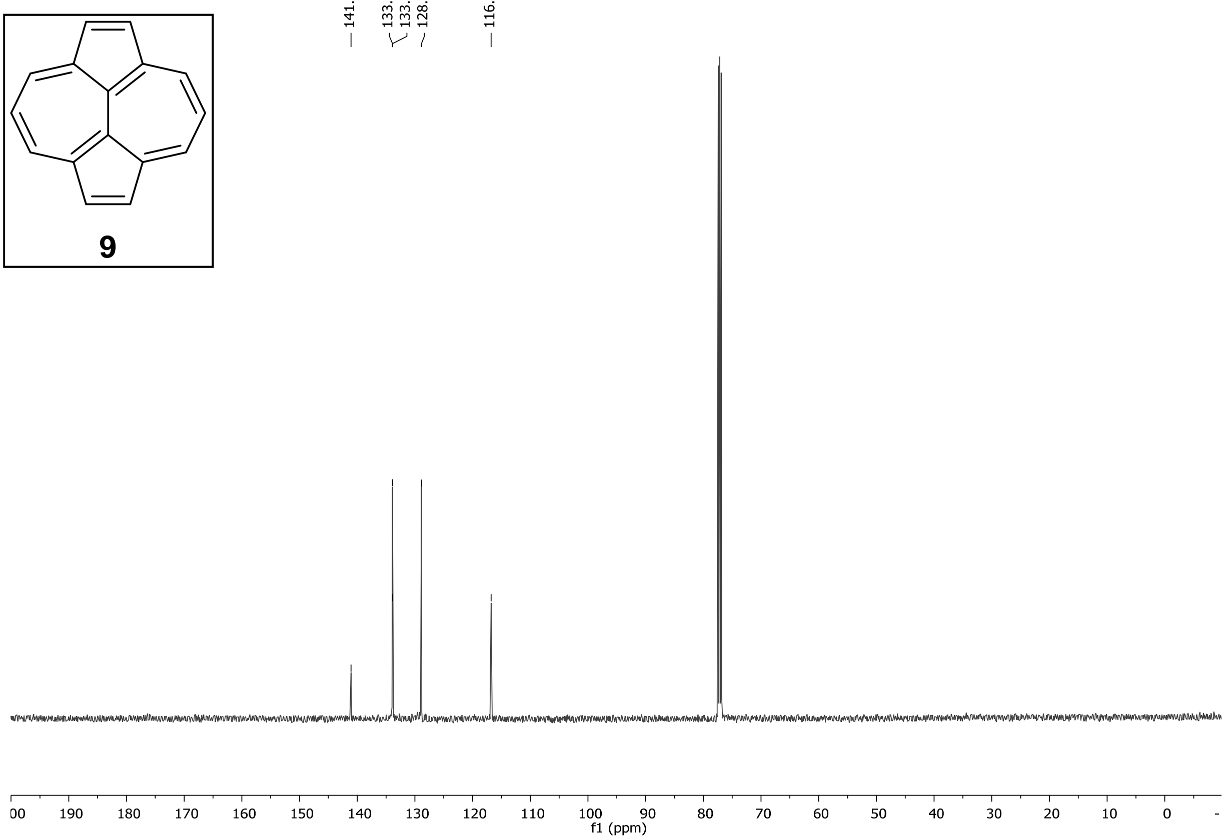

References

[1] a) A. G. Anderson, S. C. Critchlow, L. C. Andrews, R. D. Haddock, *Acta Cryst.* **1990**, C46, 439; b) A. Camerman, J. Trotter, *Acta Cryst.* **1965**, 18, 636.

[2] S. J. Clark, M. D. Segall, C. J. Pickard, P. J. Hasnip, M. I. J. Probert, K. Refson, M. C. Payne, *Z. Kristallogr. Cryst. Mater.* **2005**, 220, 567.

[3] J. P. Perdew, K. Burke, M. Ernzerhof, *Phys. Rev. Lett.* **1996**, 77, 3865.

[4] M. Schmid, H.-P. Steinrück, J. M. Gottfried, *Surf. Interface Anal.* **2014**, 46, 505.

[5] B. P. Klein, S. J. Hall, R. J. Maurer, *J. Phys.: Condens. Matt.* **2021**, 33, 154005.

[6] J. B. Birks, L. G. Christophorou, *Spectrochim. Acta* **1963**, 19, 401.

[7] K. Yamazaki, N. Niitsu, K. Nakamura, M. Kanno, H. Kono, *J. Phys. Chem. A* **2012**, 116, 11441.

[8] a) T. A. Keith, R. F. W. Bader, *Chem. Phys. Lett.* **1993**, 210, 223; b) T. A. Keith, R. F. W. Bader, *Chem. Phys. Lett.* **1992**, 194, 1; c) J. R. Cheeseman, G. W. Trucks, T. A. Keith, M. J. Frisch, *J. Chem. Phys.* **1996**, 104, 5497.

[9] P. v. R. Schleyer, C. Maerker, A. Dransfeld, H. Jiao, N. J. R. van Eikema Hommes, *J. Am. Chem. Soc.* **1996**, 118, 6317.

[10] S. Saba, D. Vrkic, C. Cascella, I. DaSilva, K. Carta, A. Kojtari, *J. Chem. Res.* **2008**, 2008, 301.

[11] J. T. Zacharia, T. Tanaka, M. Hayashi, *J. Org. Chem.* **2010**, 75, 7514.

[12] C. Jutz, E. Schweiger, *Synthesis* **1974**, 3, 193.

[13] A. G. Anderson, E. D. Daugs, L. G. Kao, J. F. Wang, *J. Org. Chem.* **1986**, 51, 2961.
